# Global tropospheric effects of aromatic chemistry with the SAPRC-11 mechanism implemented in GEOS-Chem version 9-02

**DOI:** 10.5194/gmd-12-111-2019

**Published:** 2019-01-04

**Authors:** Yingying Yan, David Cabrera-Perez, Jintai Lin, Andrea Pozzer, Lu Hu, Dylan B. Millet, William C. Porter, Jos Lelieveld

**Affiliations:** 1 Department of Atmospheric Sciences, School of Environmental Studies, China University of Geosciences (Wuhan), Wuhan, China; 2 Laboratory for Climate and Ocean–Atmosphere Studies, Department of Atmospheric and Oceanic Sciences, School of Physics, Peking University, Beijing, China; 3 Max Planck Institute for Chemistry, Atmospheric Chemistry Department, Mainz, Germany; 4 Department of Chemistry and Biochemistry, University of Montana, Missoula, MT, USA; 5 Department of Soil, Water, and Climate, University of Minnesota, St. Paul, MN, USA; 6 Department of Civil and Environmental Engineering, Massachusetts Institute of Technology, 77 Massachusetts Avenue, Cambridge, MA, USA

## Abstract

The Goddard Earth Observing System with chemistry (GEOS-Chem) model has been updated with the Statewide Air Pollution Research Center version 11 (SAPRC-11) aromatics chemical mechanism, with the purpose of evaluating global and regional effects of the most abundant aromatics (benzene, toluene, xylenes) on the chemical species important for tropospheric oxidation capacity. The model evaluation based on surface and aircraft observations indicates good agreement for aromatics and ozone. A comparison between scenarios in GEOS-Chem with simplified aromatic chemistry (as in the standard setup, with no ozone formation from related peroxy radicals or recycling of NO_x_) and with the SAPRC-11 scheme reveals relatively slight changes in ozone, the hydroxyl radical, and nitrogen oxides on a global mean basis (1 %–4 %), although remarkable regional differences (5 %–20 %) exist near the source regions. NO*_x_* decreases over the source regions and increases in the remote troposphere, due mainly to more efficient transport of peroxyacetyl nitrate (PAN), which is increased with the SAPRC aromatic chemistry. Model ozone mixing ratios with the updated aromatic chemistry increase by up to 5 ppb (more than 10 %), especially in industrially polluted regions. The ozone change is partly due to the direct influence of aromatic oxidation products on ozone production rates, and in part to the altered spatial distribution of NOx that enhances the tropospheric ozone production efficiency. Improved representation of aromatics is important to simulate the tropospheric oxidation.

## 1 Introduction

Non-methane volatile organic compounds (NMVOCs) play important roles in the tropospheric chemistry, especially in ozone production (Atkinson, 2000; Seinfeld and Pandis, 2006). Aromatic hydrocarbons such as benzene (C_6_H_6_), toluene (C_7_H_8_), and xylenes (C_8_H_10_) make up a large fraction of NMVOCs (Ran et al., 2009; Guo et al., 2006; You et al., 2008) in the atmosphere of urban and semiurban areas. They are important precursors of secondary organic aerosol (SOA), peroxyacetyl nitrate (PAN), and ozone (Kansal, 2009; Tan et al., 2012; Porter et al., 2017). In addition, many aromatic compounds can cause detrimental effects on human health and plants (Manuela et al., 2012; Sarigiannis and Gotti, 2008; Michalowicz and Duda, 2007).

Aromatics are released to the atmosphere by biomass burning as well as fossil fuel evaporation and burning (Cabrera-Perez et al., 2016; Na et al., 2004). The dominant oxidation pathway for aromatics is via reaction with the hydroxyl radical (OH, the dominant atmospheric oxidant), followed by reaction with the nitrate radical (NO_3_) (Cabrera-Perez et al., 2016; and references therein). The corresponding aromatic oxidation products could be involved in many atmospheric chemical processes, which can affect OH recycling and the atmospheric oxidation capacity (Atkinson and Arey, 2003; Calvert et al., 2002; Bejan et al., 2006; Chen et al., 2011). A realistic model description of aromatic compounds is necessary to improve our understanding of their effects on the chemistry in the atmosphere. However, up to now, few regional- or global-scale chemical transport models (CTMs) include detailed aromatic chemistry (Lewis et al., 2013; Cabrera-Perez et al., 2016).

Despite the potentially important influence of aromatic compounds on global atmospheric chemistry, their effect on global tropospheric ozone formation in polluted urban areas is less analyzed with the model simulation. The main source and sink processes of tropospheric ozone are photochemical production and loss, respectively (Seinfeld and Pandis, 2006; Monks et al., 2015; Yan et al., 2016). Observation-based approaches alone cannot provide a full picture of ozone–source attribution for the different NMVOCs. Such ozone–source relationships are needed to improve policy-making strategies to address hemispheric ozone pollution (Chandra et al., 2006). Numerical chemistry–transport models allow us to explore the importance of impacts from aromatics and to attribute observed changes in ozone concentrations to particular sources (Stevenson et al., 2006, 2013; Zhang et al., 2014). Current global CTMs reproduce much of the observed regional and seasonal variability in tropospheric ozone concentrations. However, some systematic biases can occur, most commonly an overestimation over the Northern Hemisphere (Fiore et al., 2009; Reidmiller et al., 2009; Yan et al., 2016, 2018a, b; Ni et al., 2018) due to incomplete representation of physical and chemical processes, and biases in emissions and transport, including the parameterization of small-scale processes and their feedbacks to global-scale chemistry (Chen et al., 2009; Krol et al., 2005; Yan et al., 2014, 2016).

Another motivation for the modeling comes from recent updates in halogen (bromine–chlorine) chemistry, which when implemented in the Goddard Earth Observing System with chemistry (GEOS-Chem), a global chemical transport model being used extensively for tropospheric chemistry and transport studies (Zhang and Wang, 2016; Yan et al., 2014; Shen et al., 2015; Lin et al., 2016), decrease the global burden of ozone significantly (by 14 %; 2–10 ppb in the troposphere) (Schmidt et al., 2016). This ozone burden decline is driven by decreased chemical ozone production due to halogen-driven nitrogen oxides (NO*_x_* = NO + NO_2_) loss, and the ozone decline lowers global mean tropospheric OH concentrations by 11 %. Thus, GEOS-Chem starts to exhibit low ozone biases compared to ozonesonde observations (Schmidt et al., 2016), particularly in the Southern Hemisphere, implying that some mechanisms (e.g., due to aromatics) are currently missing from the model.

A simplified aromatic oxidation mechanism has previously been employed in GEOS-Chem (e.g., Fischer et al., 2014; Hu et al., 2015), which is still used in the latest version (v12.0.0). In that simplified treatment, oxidation of benzene (B), toluene (T), and xylene (X) by OH (Atkinson, 2000) is assumed to produce first-generation oxidation products (*x*RO_2_, *x* = B, T, or X). And these products further react with hydrogen peroxide (HO_2_) or nitric oxide (NO) to produce L*x*RO_2_y (y = H or N), passive tracers which are excluded from tropospheric chemistry. Thus, in the presence of NO*_x_*, the overall reaction is aromatic + OH + NO = inert tracer. While such a simplified treatment can suffice for budget analyses of the aromatic species themselves, it does not capture ozone production from aromatic oxidation products.

In this work, we update the aromatics chemistry in GEOS-Chem based on the State-wide Air Pollution Research Center version 11 (SAPRC-11) mechanism, and use the updated model to analyze the global- and regional-scale chemical effects of the most abundant aromatics in the gas phase (benzene, toluene, xylenes) in the troposphere. Specifically, we focus on the impact on ozone formation (due to aromatics oxidation), as this is of great interest for urban areas and can be helpful for developing air pollution control strategies. Further targets are the changes to the NO*_x_* spatial distribution and OH recycling. Model results for aromatics and ozone mixing ratios are evaluated by comparison with observations from surface and aircraft campaigns in order to constrain model accuracy. Finally, we discuss the global effects of aromatics on tropospheric chemistry including ozone, NO*_x_*, and HO*_x_* (HO*_x_* = OH + HO_2_).

The rest of the paper is organized as follows. Section 2 describes the GEOS-Chem model setups, including the updates in aromatics chemical mechanism. A description of the observational datasets for aromatics and ozone is given in Sect. 3. Section 4 presents the model evaluation for aromatics based on the previously mentioned set of aircraft and surface observations, and evaluates modeled surface ozone with measurements from three networks. An analysis of the tropospheric impacts on ozone, NO*_x_*, and OH, examining the difference between models results with simplified (as in the standard model setup) and with SAPRC-11 aromatic chemistry, is presented in Sect. 5. Section 6 concludes the present study.

## 2 Model description and setup

We use the GEOS-Chem CTM (version 9–02, available at http://geos-chem.org/; last access: 28 December 2018) to interpret the importance of aromatics in tropospheric chemistry and ozone production. GEOS-Chem is a global 3-D chemical transport model for a wide range of atmospheric composition problems. It is driven by meteorological data provided by the Goddard Earth Observing System (GEOS) of the NASA Global Modeling Assimilation Office (GMAO). A detailed description of the GEOS-Chem model is available at http://acmg.seas.harvard.edu/geos/geos_chem_narrative.html(last access: 28 December 2018). Here, the model is run at a horizontal resolution of 2.5° long. × 2° lat. with a vertical grid containing 47 layers (including 10 layers of ∼ 130 m thickness, each below 850 hPa), as driven by the GEOS-5 assimilated meteorological fields. The chemistry time step is 0.5 h, while the transport time step is 15 min in the model. A non-local scheme implemented by Lin and McElroy (2010) is used for vertical mixing in the planetary boundary layer. Model convection adopts the relaxed Arakawa-Schubert scheme (Rienecker et al., 2008). Stratospheric ozone production employs the Linoz scheme (McLinden et al., 2000). Dry deposition for aromatic compounds is implemented following the scheme by Hu et al. (2015), which uses a standard resistance-in-series model (Wesely, 1989) and Henry’s law constants for benzene (0.18 M atm^−1^), toluene (0.16 M atm^−1^), and xylenes (0.15 M atm^−1^) (Sander, 1999).

### 2.1 Emissions

**Figure 1.**
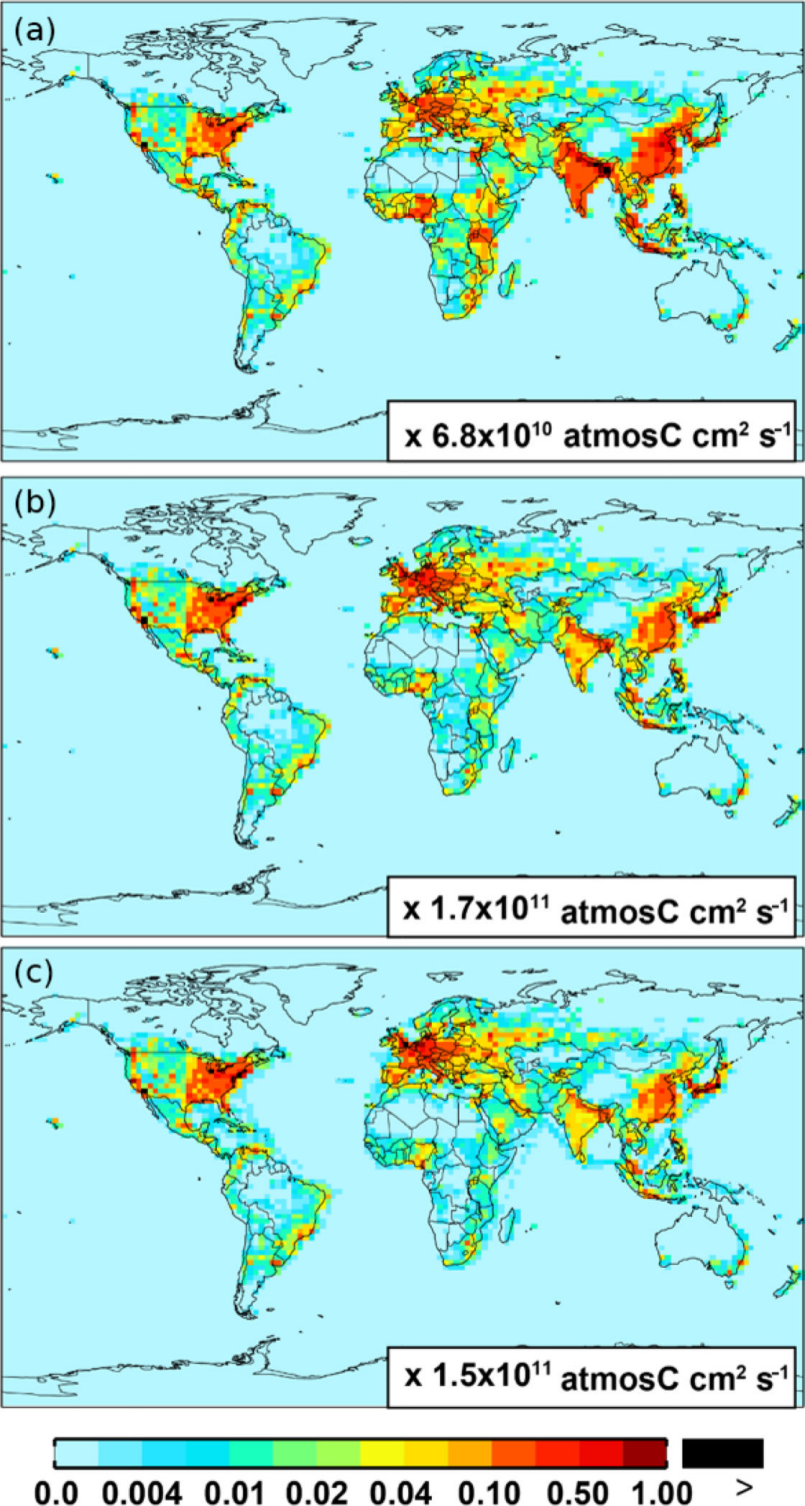
Spatial distribution of anthropogenic emissions from RETRO for benzene **(a)**, toluene **(b)**, and xylenes **(c)**, respectively.

For anthropogenic NMVOC emissions, including aromatic compounds (benzene, toluene, and xylenes), here, we use emission inventory from the RETRO (REanalysis of the TROpospheric chemical composition) (Schultz et al., 2007). The global anthropogenic RETRO (version 2; available at ftp://ftp.retro.enes.org/) inventory includes monthly emissions for 24 distinct chemical species during 1960–2000 with a resolution of 0.5° long. × 0.5° lat. (Schultz et al., 2007). It is implemented in GEOS-Chem by regridding to the model resolution (2.5° long. × 2.0° lat.). Emission factors in RETRO are calculated on account of economic and technological considerations. In order to estimate the time dependence of anthropogenic emissions, RETRO also incorporates behavioral aspects (Schultz et al., 2007). The implementation of the monthly RETRO emission inventory in GEOS-Chem is described by Hu et al. (2015), which linked the RETRO species into the corresponding model tracers. Here, the model speciation of xylenes includes m-xylene, p-xylene, o-xylene, and ethylbenzene (Hu et al., 2015). The most recent RETRO data (for 2000) are used for the GEOS-Chem model simulation, and the calculated annual global anthropogenic NMVOCs are ∼ 71 Tg C. On a carbon basis, the global aromatics (benzene plus toluene plus xylenes) source accounts for ∼ 23 % (16 Tg C) of the total anthropogenic NMVOCs. Figure 1 shows the spatial distribution of anthropogenic emissions for benzene, toluene, and xylenes, respectively. Anthropogenic benzene emissions in Asia (mainly over eastern China and India) are larger than those from other source regions (e.g., over Europe and the eastern US).

Global NO*_x_* anthropogenic emissions are taken from the EDGAR (Emissions Database for Global Atmospheric Research) v4.2 inventory. The global inventory has been replaced by regional inventories in China (MEIC, base year 2008), Asia (excluding China; INTEX-B, 2006), the US (NEI05, 2005), Mexico (BRAVO, 1999), Canada (CAC, 2005), and Europe (EMEP, 2005). Details on these inventories and on the model NO*_x_* anthropogenic emissions are shown in Yan et al. (2016).

Biomass burning emissions of aromatics and other chemical species (e.g., NO*_x_*) in GEOS-Chem are calculated based on the monthly Global Fire Emission Database version 3 (GFED3) inventory (van der Werf et al., 2010). Natural emissions of NO*_x_* (by lightning and soil) and of biogenic NMVOCs are calculated online by parameterizations driven by model meteorology. Lightning NO*_x_* emissions are parameterized based on cloud-top heights (Price and Rind, 1992) and are further constrained by the lightning flash counts detected from satellite instruments (Murray et al., 2012). Soil NO*_x_* emissions are described in Hudman et al. (2012). Biogenic emissions of NMVOCs are calculated by MEGAN (Model of Emissions of Gases and Aerosols from Nature) v2.1 with the hybrid algorithm (Guenther et al., 2012).

### 2.2 Updated aromatic chemistry

In the GEOS-Chem model setup, the current standard chemical mechanism with simplified aromatic oxidation chemistry is based on Mao et al. (2013), which is still the case for the latest version (v12.0.0). As mentioned in the introduction, this simplified mechanism acts as strong sinks of both HO*_x_* and NO*_x_*, because no HO*_x_* is regenerated in this reaction, and NO is consumed without regenerating NO_2_. However, it is reasonably well established that aromatics tend to be radical sources, forming highly reactive products that photolyze to form new radicals, and regenerating radicals in their initial reactions (Carter, 2010a, b; Carter and Heo, 2013). A revised mechanism that takes the general features of aromatics mechanisms into account would be much more reactive, given the reactivity of the aromatic products.

This work uses a more detailed and comprehensive aromatics oxidation mechanism: the SAPRC-11 aromatics chemical mechanism. SAPRC-11 is an updated version of the SAPRC-07 mechanism (Carter and Heo, 2013) to give better simulations of recent environmental chamber experiments. The SAPRC-07 mechanism underpredicted NO oxidation and O_3_ formation rates observed in recent aromatic– NO*_x_* environmental chamber experiments. The new aromatics mechanism, designated SAPRC-11, is able to reproduce the ozone formation from aromatic oxidation that is observed in almost all environmental chamber experiments, except for higher (> 100 ppb) NO*_x_* (Carter and Heo, 2013). Table S1 in the Supplement lists new model species in addition to those in the standard GEOS-Chem model setup. Table S2 lists the new reactions and rate constants. In this mechanism, the tropospheric consumption process of aromatics is mainly the reaction with OH.

As discussed by Carter (2010a, b), aromatic oxidation has two possible OH reaction pathways: OH radical addition and H-atom abstraction (Atkinson, 2000). In SAPRC-11, taking toluene as an example in Table S2, the reactions following abstraction lead to three different formation products: an aromatic aldehyde (represented as the BALD species in the model), a ketone (PROD2), and an aldehyde (RCHO). The largest yield of toluene oxidation is the reaction after OH addition of aromatic rings. The OH–aromatic adduct is the reaction with O_2_ to form an OH–aromatic–O_2_ adduct or HO_2_ and a phenolic compound (further consumed by reactions with OH and NO_3_ radicals). The OH–aromatic–O_2_ adduct further undergos two competing unimolecular reactions to ultimately form OH, HO_2_, an α-dicarbonyl – such as glyoxal (GLY), methylglyoxal (MGLY), or biacetyl (BACL) – a monounsaturated dicarbonyl co-product (AFG1, AFG2, the photoreactive products) and a di-unsaturated dicarbonyl product (AFG3, the non-photoreactive products) (Calvert et al., 2002).

Formed from the phenolic products, the SAPRC-11 mechanism includes species of cresols (CRES), phenol (PHEN), xylenols and alkyl phenols (XYNL), and catechols (CATL). Due to their different SOA and ozone formation potentials (Carter et al., 2012), these phenolic species are represented separately. Relatively high yields of catechol (CATL) have been observed in the reactions of OH radicals with phenolic compounds. Furthermore, their subsequent reactions are believed to be important for SOA and ozone formation (Carter et al., 2012).

### 2.3 Simulation setups

In order to investigate the global chemical effects of the most commonly emitted aromatics in the troposphere, two simulations were performed: one with the ozone related aromatic chemistry updates from SAPRC-11 (the SAPRC case) and the other with simplified aromatic chemistry as in the standard setup (the Base case). Both simulations (Base and SAPRC) at 2.5° long, × 2° lat. are conducted from July 2004 to December 2005, allowing for a 6-month spin-up for our focused analysis over the year 2005 for comparison to the available observations (Sect. 3). Initial conditions of chemicals are regridded from a simulation at 5° long, × 4° lat. started from 2004 with another spin-up run from January to June 2004. For comparison with aromatics observations over the US in 2010–2011 (Sect. 3), we extend the simulations from July 2009 to December 2011 with July-December 2009 as the spin-up period.

## 3 Aromatics and ozone observations

We use a set of measurements from surface and aircraft campaigns to evaluate the model-simulated aromatics and ozone.

### 3.1 Aromatic aircraft observations

For aromatics, we use airborne observations from CALNEX (California; May/June 2010) aircraft study. A proton transfer reaction quadrupole mass spectrometer (PTR-MS) was used to measure mixing ratios of aromatics (and an array of other primary and secondary pollutants) during CALNEX. Measurements are gathered mostly on a 1 s timescale (approximately 100m spatial resolution), which permits sampling of the source regions and tracking subsequent transport and transformation throughout California and the surrounding regions. Further details of the CALNEX campaign, including the flight track, time frame, location, and instrument, are shown in Hu et al. (2015) and https://www.esrl.noaa.gov/csd/ projects/calnex (last access: 28 December 2018). For comparison to the model results, we averaged the high temporal-spatial resolution observations to the model resolution.

We also employ vertical profiles obtained in 2005 from the CARIBIC (Civil Aircraft for Regular Investigation of the atmosphere Based on an Instrument Container) project, which conducts atmospheric measurements aboard a commercial aircraft (Lufthansa A340–600) (Brenninkmeijer et al., 2007; Baker et al., 2010). CARIBIC flights fly away from Frankfurt, Germany, on the way to North America, South America, India, and east Asia. Measurements are available in the upper troposphere (50 % on average) and lower stratosphere (50 %) (UTLS) at altitudes between 10 and 12 km. To evaluate our results, measurements are averaged to the model output resolution. Vertically, results from GEOS-Chem model simulations at the 250 hPa level are used to compare with observations between 200 and 300 hPa. Then, the annual means of observations and model data sampled along the flight tracks are used in the comparison.

### 3.2 Aromatic surface measurements

To evaluate the ground-level mixing ratios of benzene, toluene, and xylenes as well as their seasonal cycles, surface observations of aromatics are collected from two networks (EMEP, data available at http://www.nilu.no/projects/ccc/emepdata.html; last access: 28 December 2018, and the European Environmental Agency – EEA, data available at http://www.eea.europa.eu/data-and-maps/data/airbase-the-european-air-quality-database-8; last access: 28 December 2018, both for the year 2005) over Europe and the KCMP tall tower dataset (data available at https://atmoschem.umn.edu/data; last access: 28 December 2018, for the year 2011) over the US.

EMEP, which aims to investigate the long-range transport of air pollution and the flux through geographic boundaries (Torseth et al., 2012), locates measurement sites in locations where there are minimal local impacts; thus, consequently, the observations could represent the feature of large regions. EMEP has a daily resolution with a total of 14 stations located in Europe for benzene, 12 stations for toluene, and 8 stations for xylenes (Table 1). Here, we use the monthly values calculated from the database to evaluate monthly model results. Note that measurement speciation of xylenes (o-xylene, m-xylene, and p-xylene) in EMEP network does not exactly correspond with the model speciation of xylenes (m-xylene, p-xylene, o-xylene and ethylbenzene) (Hu et al., 2015). The speciation assumption probably can partly account for the xylene model–measurement discrepancy seen in Sect. 4.

EEA provides observations from a large number of sites over urban, suburban, and background regions (EEA, 2014). However, here, we use only rural background sites to do model comparison, as in Cabrera-Perez et al. (2016), because the model horizontal scale cannot simulate direct traffic or industrial influence. This leads to 22 stations available for benzene and 6 stations for toluene. Further details of the sites and location information of EEA (and EMEP) used here are described in Cabrera-Perez et al. (2016). For comparison, annual means for individual sites have been used.

The KCMP tall tower measurements (at 44.69° N, 93.07° W; Minnesota, US) have been widely used for studies of surface fluxes of tropospheric trace species and land-atmosphere interactions (Kim et al., 2013; Hu et al., 2015; Chen et al., 2018). A suite of NMVOCs including aromatics were observed at the KCMP tower during 2009–2012 with a high-sensitivity PTR-MS, sampling from a height of 185 m a.g.l. (above ground level). We averaged the hourly observations of benzene, toluene, and C_8_ (xylenes plus ethylbenzene, here consistent with the model speciation) aromatics to monthly values and then used them for our model evaluation. Monthly mean simulations at the 990 hPa level (∼ 190 m) are used for comparison.

### 3.3 Ozone observations

Ozone observations are taken from the database of the World Data Centre for Greenhouse Gases (WDCGG, data available at http://ds.data.jma.go.jp/gmd/wdcgg/cgi-bin/wdcgg/catalogue.cgi; last access: 28 December 2018), and the Chemical Coordination Centre of EMEP (EMEP CCC). These networks contain hourly ozone measurements over a total of 194 background sites in remote environments. We use monthly averaged observations of surface ozone in 2005 to examine the simulated surface ozone from the GEOS-Chem model. Simulated ozone from the lowest layer (centered at ∼ 65 m) is sampled from the grid cells corresponding to the ground sites.

## 4 Evaluation of simulated aromatics and ozone

In this section, the SAPRC model simulation results of aromatics (benzene, toluene, xylenes, and C_8_ aromatics) and ozone from GEOS-Chem are evaluated with observations. Table 1 summarizes the statistical comparison between measured and simulated concentrations over the monitoring stations described in Sect. 3. For the statistical calculations, GEOS-Chem simulation results have been sampled along the geographical locations of the measurements. Table 1 includes the number of locations and time resolutions. The number of sites in EEA for xylenes is only two; thus, we do not include their comparison results in Table 1 due to the lack of representativeness.

### 4.1 Surface-level aromatics

For the aromatics near the surface mixing ratios over Europe, observed mean benzene (194.0 ppt for EEA and 166.4 ppt for EMEP) and toluene (240.3 ppt for EEA and 133.1 ppt for EMEP) mixing ratios are higher than observed mean xylene concentrations (42.3 ppt for EMEP). In general, the model underestimates EEA and EMEP observations of benzene (by 34 % on average) and toluene (by 20 % on average). For benzene, the model results systematically underestimate the annual means (36 %) compared to the EMEP database, consistent with the model underestimate of the EEA dataset (32 %). The model underestimate for toluene compared to the EMEP dataset (15 %) is smaller than that relative to the EEA measurements (25 %). The simulation overestimates the xylene measurements in EMEP by a factor of 1.9, in part because the model results include ethylbenzene but the observations do not (see Sect. 3.2). The fact that the anthropogenic RETRO emissions (for the year 2000) do not correspond to the year of measurement (2005) may contribute to the above model–measurement discrepancies. Anthropogenic aromatics emissions are reported to have significant changes in emissions and their distributions over the decade by EDGAR v4.3.2 (Crippa et al., 2018; http://eccad.aeris-data.fr/B/#DatasetPlace:EDGARv4.3.2$DOI; last access: 28 December 2018). It shows that the total aromatics emissions from anthropogenic sources are enhanced by 5 % (2005) and 14 % (2011) compared to the year 2000. The model bias would be partly benefit from this emission increase with enhanced modeled mixing ratios of benzene and toluene.

The modeled spatial variability of aromatics (with standard deviations of 32.1–66.8 ppt) is 18 %–73 % lower than that of the EMEP and EEA observations (41.9–118.4 ppt), probably due to the coarse model resolution. The spatial variability in benzene (46 %–73 % lower) is the most strongly underestimated among the three aromatic species. Unlike benzene, simulated concentrations of toluene show a larger standard deviation (66.8 ppt) than the EEA measurements (59.4 ppt), indicating larger simulated spatial variability. Simulation results are thus poorly spatially correlated with observations (*R* = 0:41–0.49). However, the temporal variability of aromatics is well captured by GEOS-Chem with the correlations above 0.7 for most stations.

**Figure 2.**
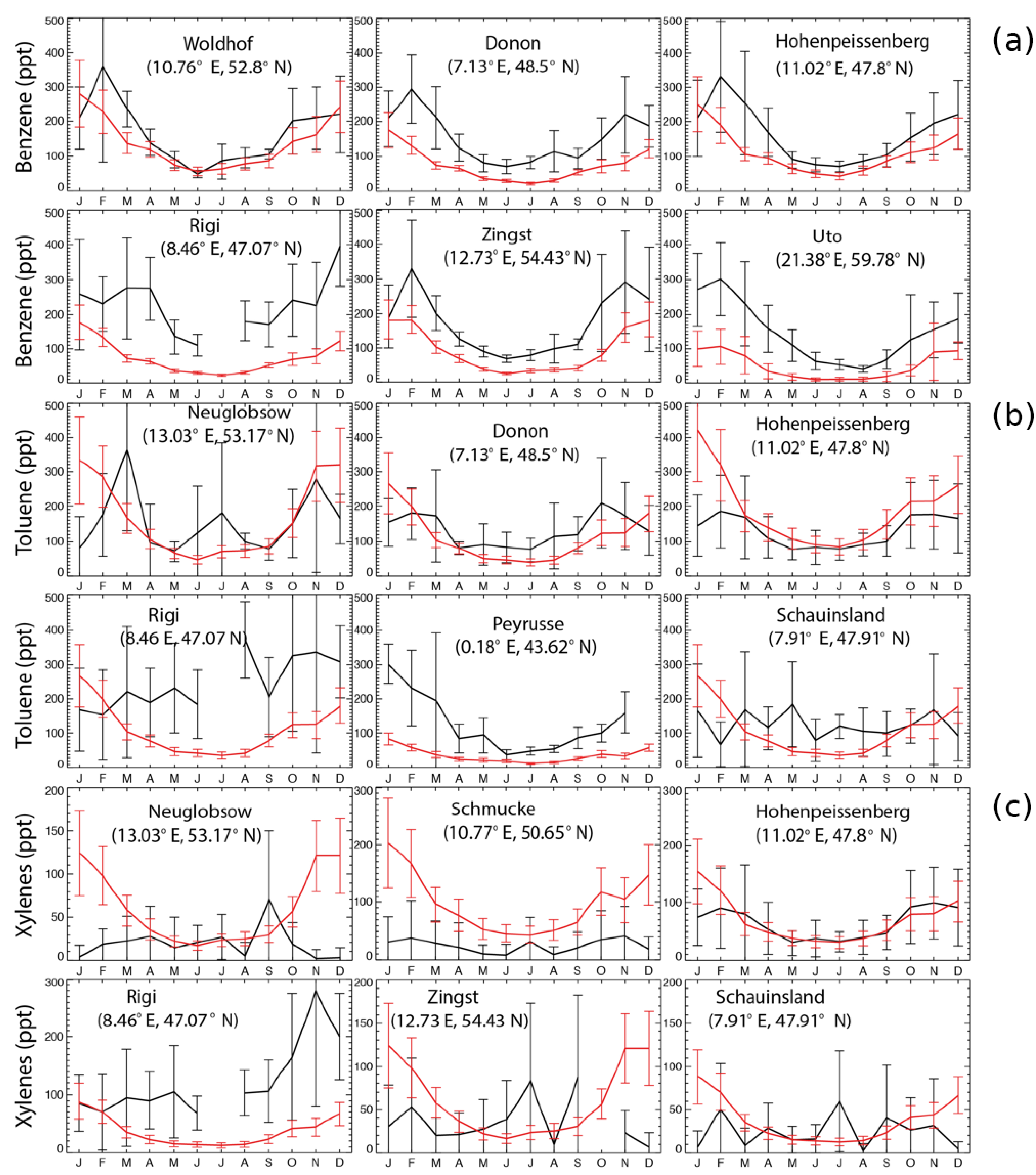
Monthly average EMEP observations (in black) of benzene **(a)**, toluene **(b)**, and xylenes **(c)** at six different locations for the year 2005, as well as the model results in the SAPRC simulation (in red), both in ppt. Error bars show the standard deviations.

Figure 2 shows a comparison of model results with observations at six stations for benzene, toluene, and xylenes, respectively, following Cabrera-Perez et al. (2016). The sites are chosen as the first six stations with largest amount of data. Model results reproduce the annual cycle at the majority of sites. Aromatics are better simulated in summer than in winter. This feature has been previously found for the climate–chemistry ECHAM/MESSy Atmospheric Chemistry (EMAC) model for aromatics (Cabrera-Perez et al., 2016) and simpler NMVOCs (Pozzer et al., 2007). In addition, the measurements show larger standard deviations than the GEOS-Chem simulations, with the ratios between the observed and the simulated standard deviations being 2–11.

**Figure 3.**
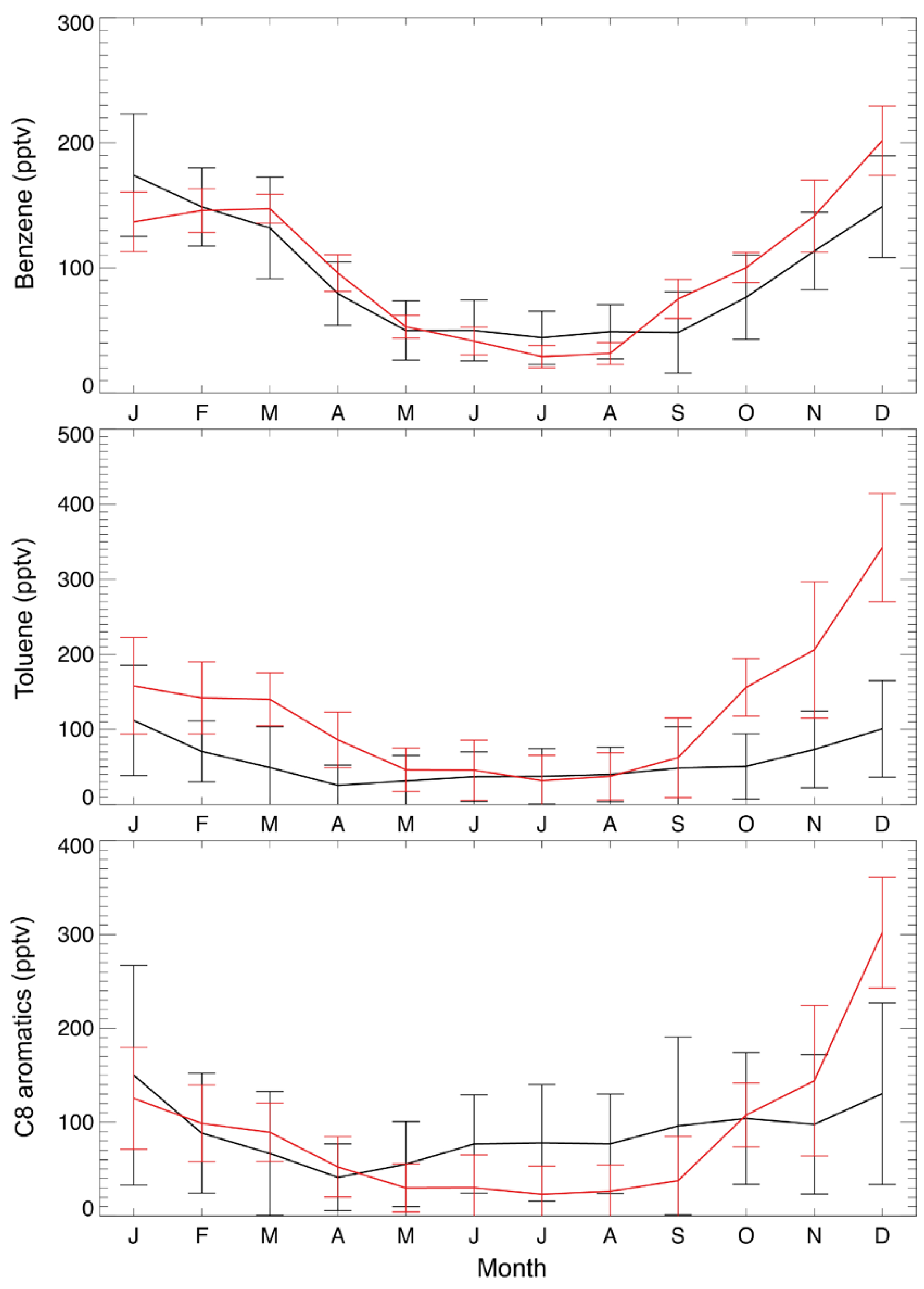
Monthly average KCMP tall tower observations (in black) of benzene, toluene, and C_8_ (xylenes plus ethylbenzene) aromatics in the year 2011 and the model results in the SAPRC simulation (in red). Error bars show the standard deviations.

Over the US, annual mean observed concentrations at the KCMP tall tower are 91.5 ppt for benzene, 56.7 ppt for toluene, and 90.3 ppt for C8 aromatics (Table 1). The model biases for benzene (8.4 ppt; 9.2 %) and C_8_ aromatics (−1:4 ppt; −1:6 %) are much lower than that for toluene (64.5 ppt; 114 %). Figure 3 further shows the observed and simulated monthly averaged concentrations of benzene, toluene, and C_8_ aromatics. The SAPRC simulation reproduces their seasonal cycles, with higher concentrations in winter and lower mixing ratios in summer, consistent with Hu et al. (2015). The model–observation correlations are 0.89, 0.78, and 0.65 for monthly benzene, toluene, and C_8_ aromatics, respectively. The large overestimation of modeled toluene is mainly due to simulated high mixing ratios during the cold season (Fig. 3, October to March).

### 4.2 Tropospheric aromatics

Table 1 shows that, in the UTLS, both CARIBIC-observed (16 ppt) and GEOS-Chem-modeled (12.3 ppt) benzene mixing ratios are higher than toluene concentrations (3.6 ppt for CARIBIC and 1.5 ppt for GEOS-Chem). For benzene, the model underestimations appear to be smaller in the free troposphere (with an underestimation by 23 %) than at the surface (36 % for EMEP and 32 % for EEA). In contrast to benzene, annual mean concentrations of toluene are underestimated by 58 % in the UTLS. The geographical variability of benzene is larger than that for toluene (with standard deviation of 4.2 ppt versus 0.7 ppt in model and 15.8 ppt versus 7.5 ppt in observation), probably because of the shorter lifetime of benzene (between several hours and several days; http://www.nzdl.org/gsdlmod?a=p&p=home&l=en&w=utf-8; last access: 28 December 2018), in combination with the lower concentrations in the UTLS for toluene. The model results show smaller spatial variability than the observations. This underestimation for spatial variability in the free troposphere (over 70 %) is higher than that at the surface (not shown).

**Figure 4.**
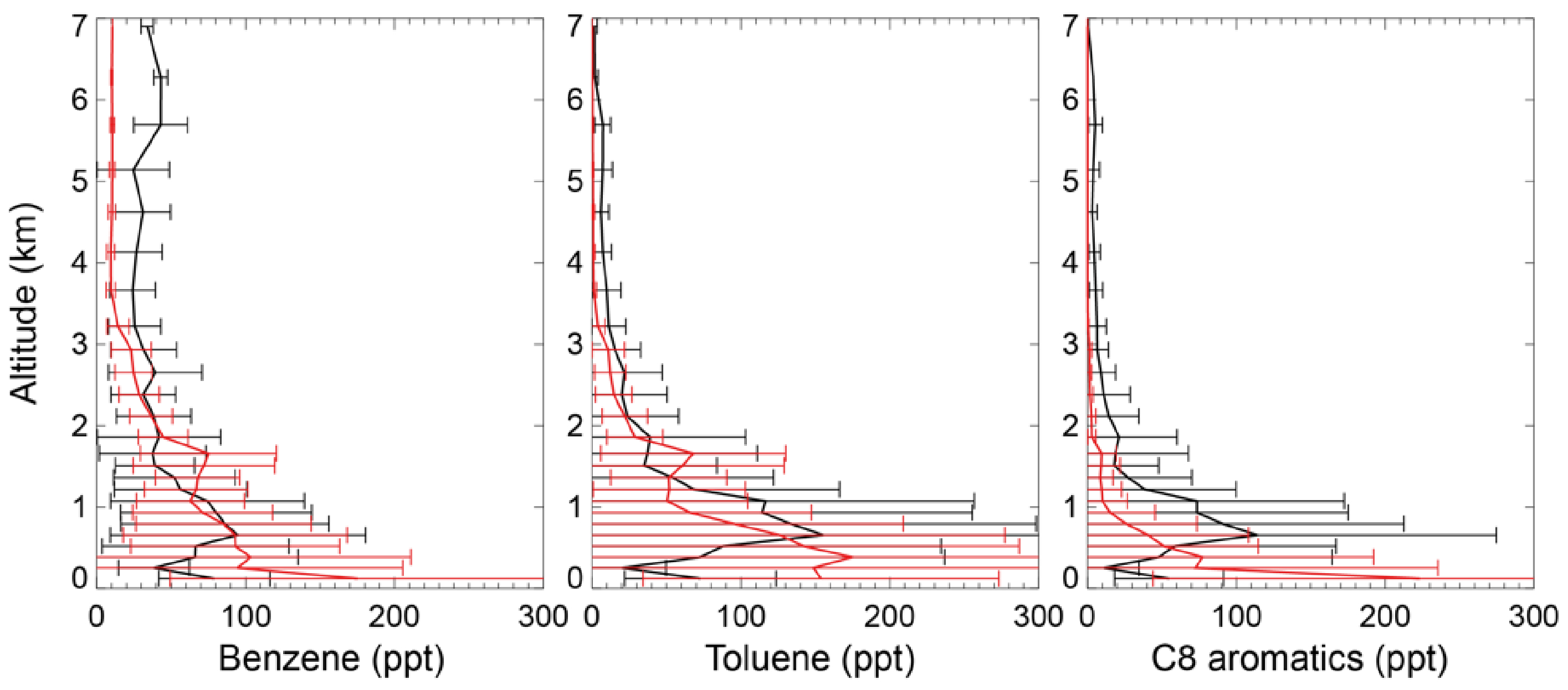
Measured (black) and simulated (red for the SAPRC case) vertical profiles of aromatics in May/June 2010 for the CALNEX campaigns. Model results are sampled at times and locations coincident to the measurements. Horizontal lines indicate the standard deviations.

The black lines in Fig. 4 show the tropospheric aromatics profiles during the CALNEX campaign. The measured values peak at an altitude of 0.6–0.8 km, with concentrations decreasing at higher altitudes. Although the concentrations in the lower troposphere for benzene (40–100 ppt below 2 km) are lower than mixing ratios for toluene (70– 160 ppt below 2 km) and C_8_ aromatics (50–120 ppt below 2 km), the benzene mixing ratios (> 30 ppt) in the free troposphere are much higher than those of toluene and C_8_ aromatics (< 10 ppt). The different profile shapes in the lower troposphere for benzene, toluene, and C_8_ aromatics are mainly due to their different emissions and lifetime. The SAPRC simulation (red lines in Fig. 4) captures the general vertical variations of CALNEX benzene and toluene, with statistically significant model–observation correlations of 0.74 and 0.65 for benzene and toluene, respectively. The model generally overestimates the measured C_8_ aromatics below 0.5 km, albeit with an underestimation above 0.5 km, with lower model-observation correlation of 0.37. This overestimation below 0.5 km is also seen for benzene and toluene. The modeled overly rapid aromatics drop-off with altitude probably implies the modeled aromatics lifetime is short.

### 4.3 Surface ozone

**Figure 5.**
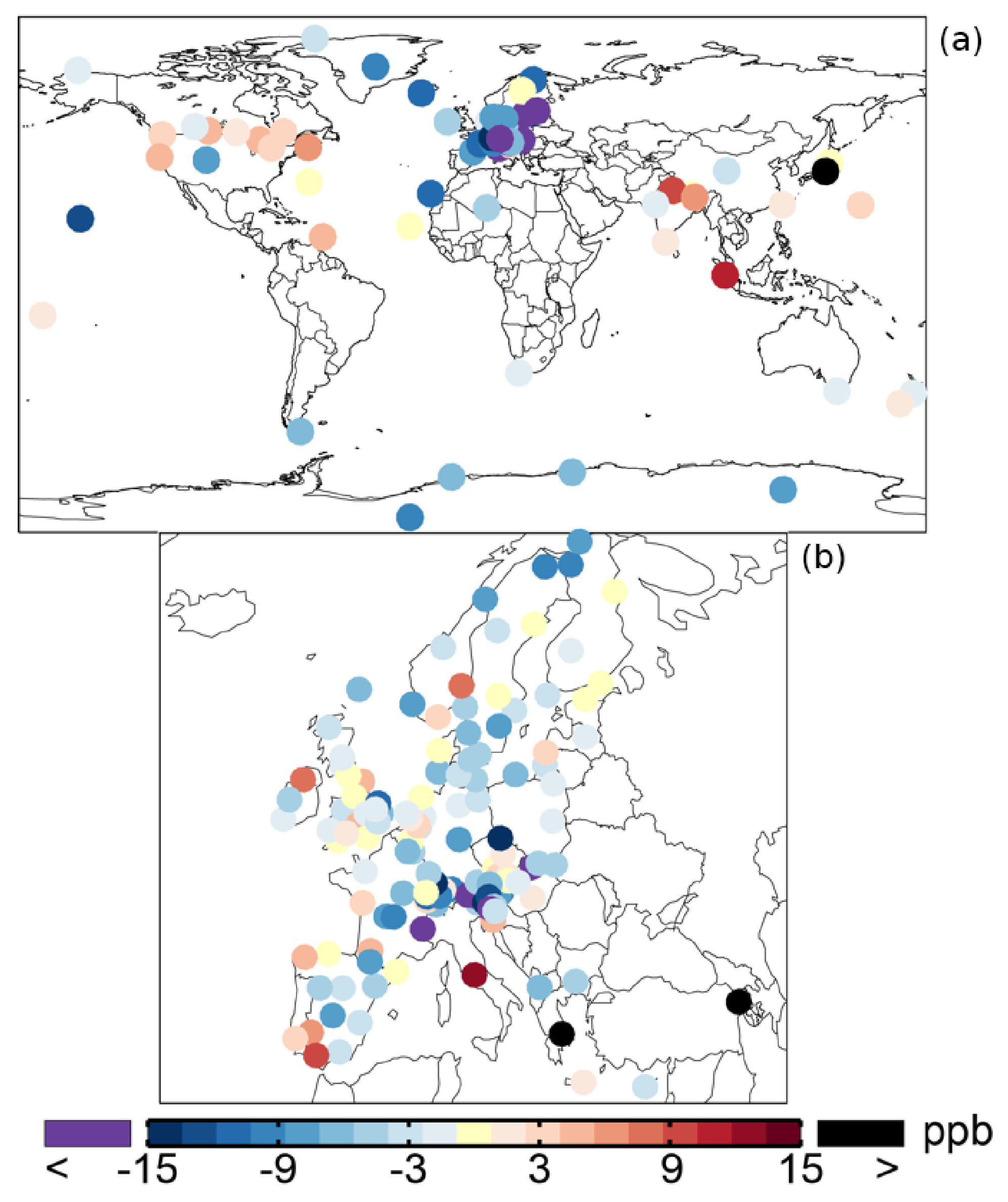
Annual mean model biases for surface ozone in the SAPRC simulation, with respect to measurements from WD-CGG **(a)** and EMEP **(b)** networks.

Table 1 shows an average ozone mixing ratio of 34.1 ppb in 2005 over the regional background WDCGG sites. The annual mean ozone mixing ratios are lower over Europe (from the EMEP dataset), about 30.6 ppb. The SAPRC simulation tends to underestimate the mixing ratios over the sites of Europe and background regions with biases of −2:9 and −5:5 ppb, respectively. Figure 5 shows the spatial distribution of the annual mean model biases with respect to the measurements. Unlike the modeled surface aromatics, the simulated ozone spatial variability can be either slightly lower or higher than the observed variability, depending on the compared database: the standard deviation is 12.8 ppb (simulated) versus 14.2 ppb (observed) for WDCGG sites, 13.2 ppb versus 10.3 ppb for EMEP sites. The temporal variability (temporal correlations of 0.68–0.72) is better captured by the model than the spatial variability (spatial correlations of 0.52–0.54).

## 5 Global effects of aromatic chemistry

This section compares the Base and SAPRC simulations to assess to which extent the updated mechanism for aromatics affect the global simulation of ozone, HO*_x_*, and individual nitrogen species. Our focus here is on the large-scale impacts.

### 5.1 NO_y_ species

**Figure 6.**
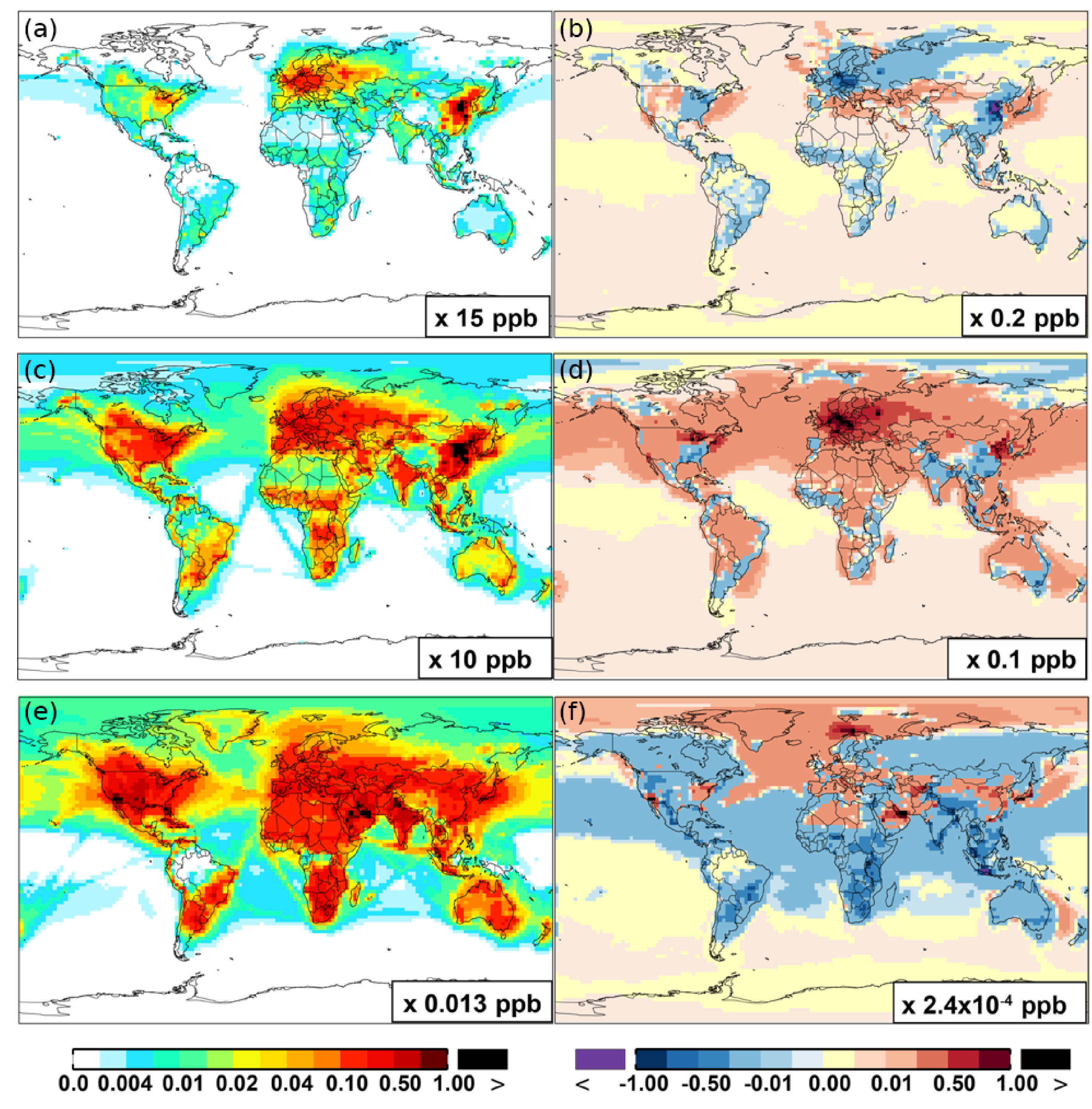
**(a, c, e)** Modeled spatial distributions of annual mean surface NO **(a, b)**, NO_2_ **(c, d)**, and NO_3_ **(e, f)** simulated in the Base case for the year 2005. **(b, d, f)** The respective changes from Base to SAPRC.

Figure 6 and Table 2 show the changes from Base to SAPRC in annual average surface NO mixing ratios. A decrease in NO is apparent over NO*_x_* source regions, e.g., by approximately 0.15 ppb (∼ 20 %) over much of the US, Europe and China (Fig. 6). In contrast, surface NO increases at locations downwind from NO*_x_* source regions (up to ∼ 0:1 ppb or 20 %), including the oceanic area off the eastern US coast, the marine area adjacent to Japan, and the Mediterranean area. The change is negligible (by −0:2 %) for the annual global mean surface NO (Table 2). Seasonally, the decrease in spring, summer, and fall is compensated partly by the increase in winter (Table 2). This winter increase versus decline in other seasons is probably attributed to the weakened photochemical reactions involving NO*_x_* in winter.

**Figure 7.**
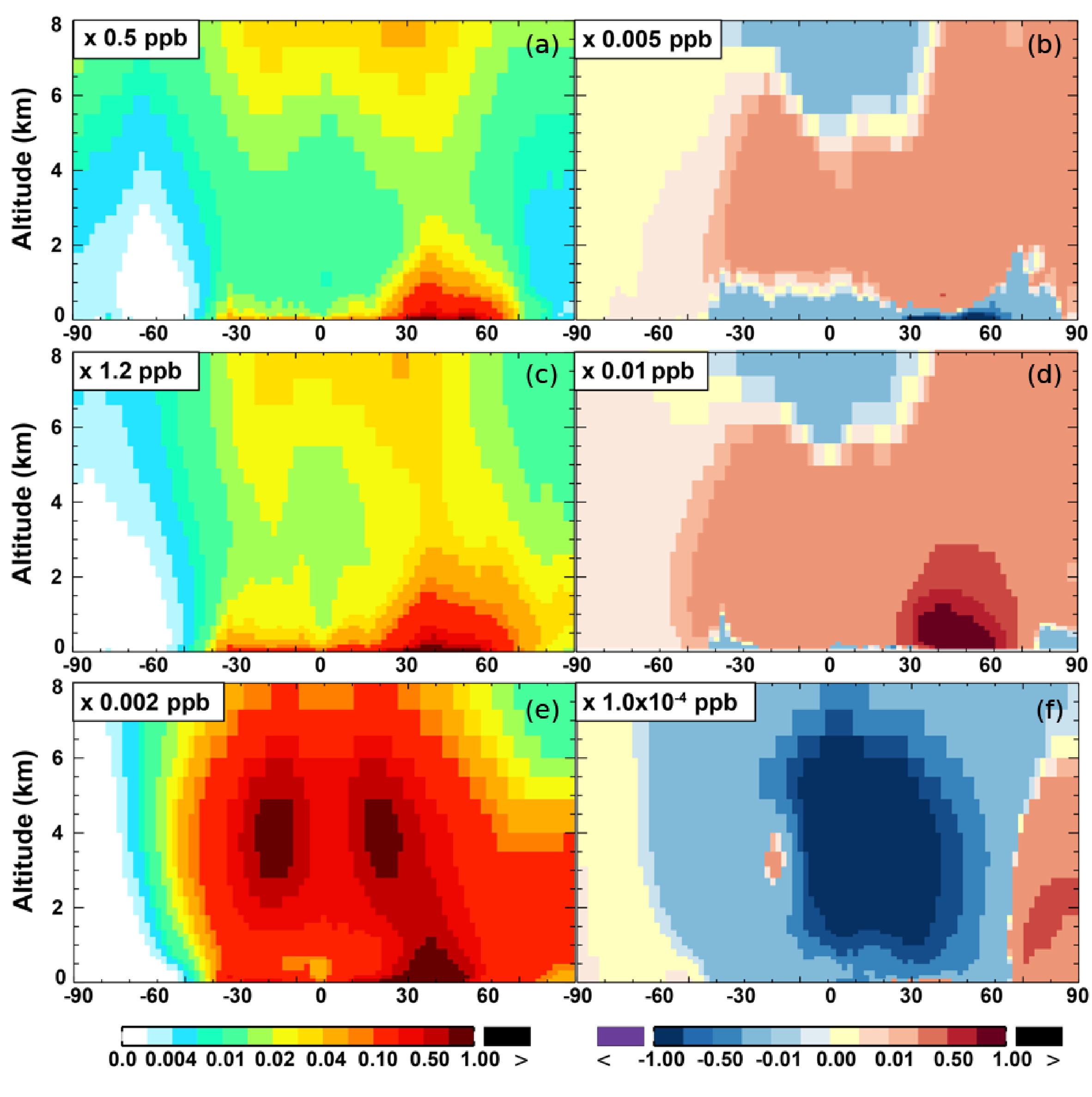
**(a, c, e)** Modeled zonal average latitude-altitude distributions of annual mean NO **(a, b)** and NO_2_ **(c, d)**, and NO_3_ **(e, f)** simulated in the Base scenario for the year 2005. **(b, d, f)** The respective changes from Base to SAPRC.

The zonal average results in Fig. 7 show a clear decline in NO in the planetary boundary layer, in contrast to significant increases in the free troposphere, from Base to SAPRC. The free tropospheric NO increases are about the same from 30° S to 90° N, with an annual average enhancement up to 5 % (Fig. 7), and are particularly large in winter (up to 10 %, not shown). For the whole troposphere, the average NO increases by 0.6 % from Base to SAPRC (Table 2).

**Figure 8.**
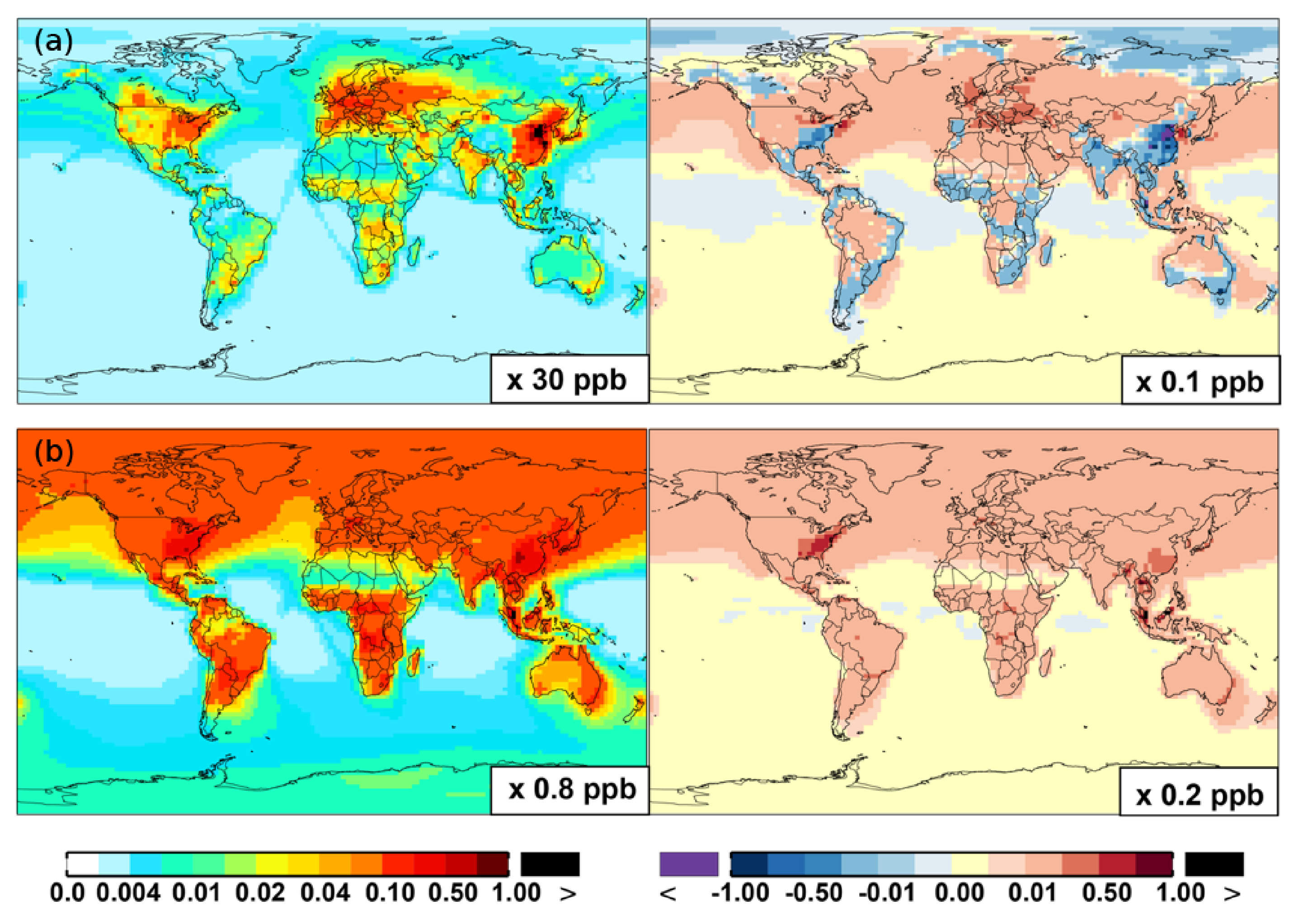
Same as Fig. 6 but for NO*_x_* **(a)** and PAN **(b)**.

**Figure 9.**
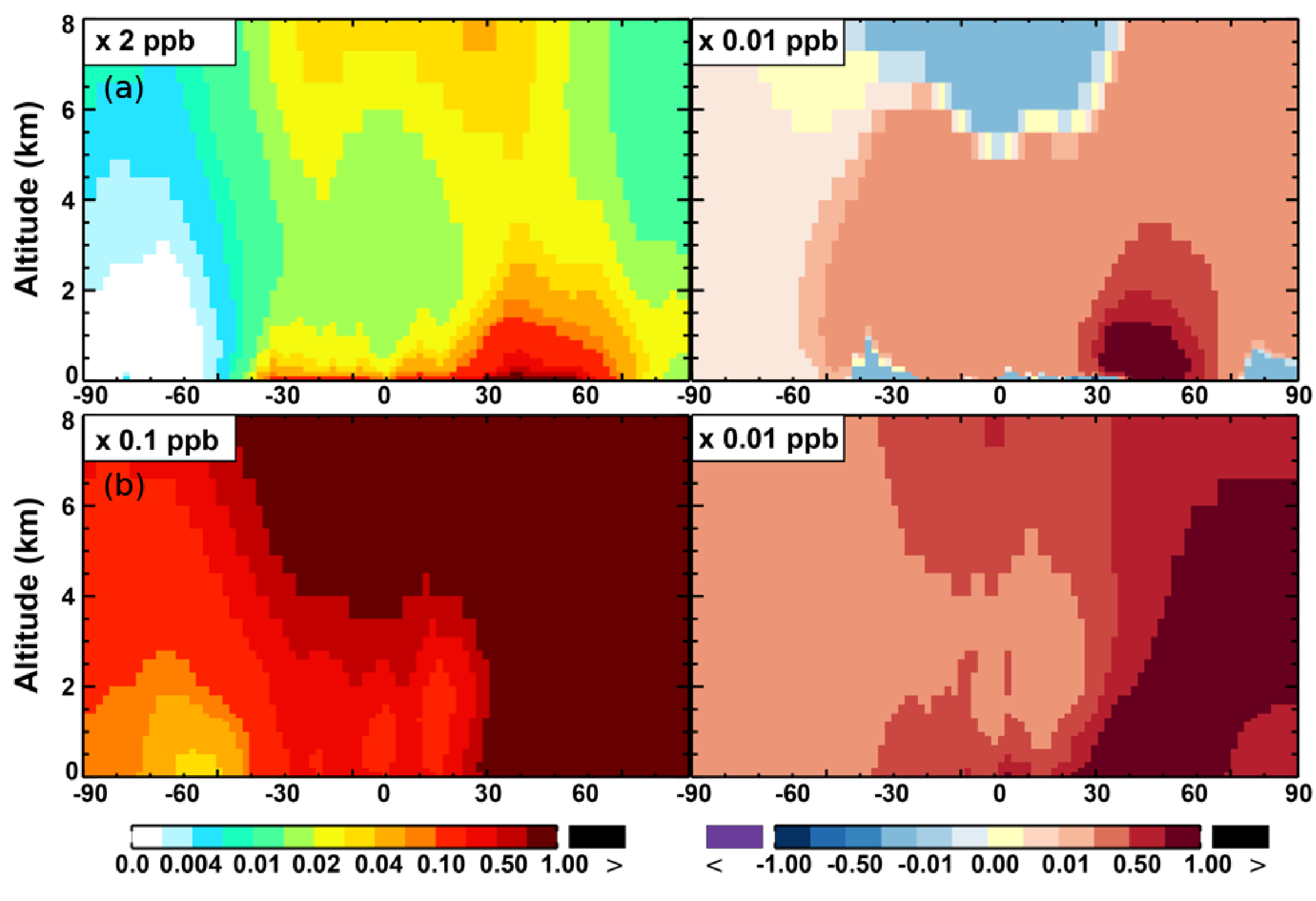
Same as Fig. 7 but for NO*_x_* **(a)** and PAN **(b)**.

Figure 6 shows that simulated surface NO_2_ mixing ratios in the SAPRC scenario are enhanced over most locations across the globe, in comparison with the Base simulation. Over the source regions, the changes are mixed, with increases in some highly NO*_x_* polluted regions (by up to 10 %) and decreases in other polluted regions. On a global mean basis, NO_2_ is increased (by 2.1 % in the free troposphere and 1.0 % at the surface; Table 2), due mainly to the recycling of NO*_x_* from PAN associated with the aromatics, and the reactions of oxidation products from aromatics with NO or NO_3_ (primarily) to form NO_2_ and HO_2_. Combing the changes in NO and NO_2_ means that the total NO_x_ mixing ratios decrease in source regions but increase in the remote free troposphere (Figs. 8 and 9).

The NO_3_ mixing ratios decrease at the global scale (−4:1 % on average in the troposphere; Fig. 7 and Table 2) in the SAPRC simulation, except for an enhancement in surface NO_3_ over the northern polar regions and most polluted areas like the eastern US, Europe and eastern China (Fig. 6). The NO_3_ global decreases are mainly due to the consumption of NO_3_ by reaction with the aromatic oxidation products. However, the NO_3_ regional increases are probably caused by the enhanced regional atmospheric oxidation capacity.

Table 2 shows that nitric acid (HNO_3_) increases in the SAPRC simulation, both near the surface (by approximately 1.1 %) and in the troposphere (by 0.3 %). The enhancement in HNO_3_ appears uniformly over most continental regions in the Northern Hemisphere (not shown), due to the promotion of direct formation of HNO_3_ from aromatics in the SAPRC simulation.

### 5.2 OH and HO_2_

**Figure 10.**
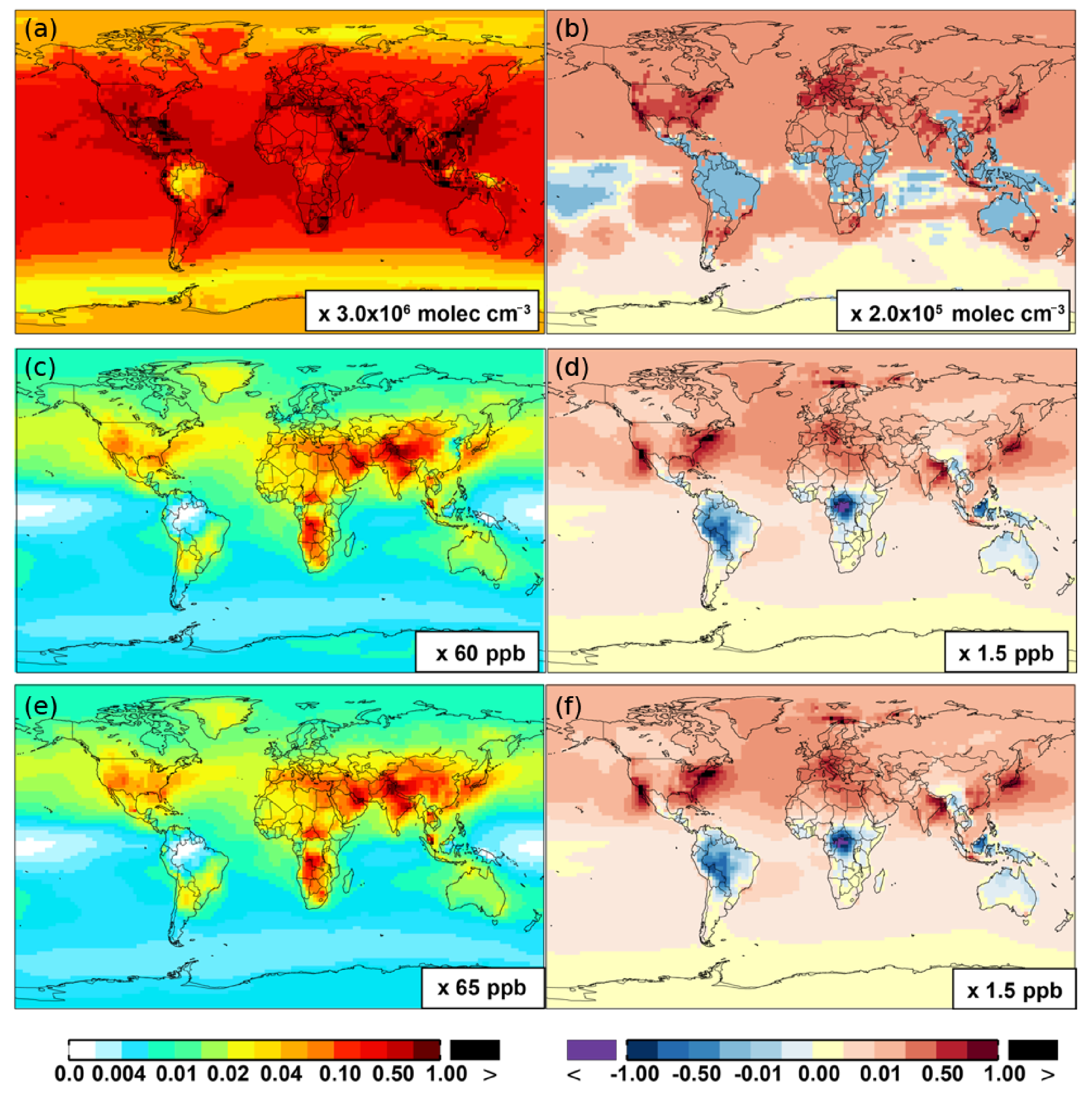
Same as Fig. 6 but for OH **(a, b)**, O_3_ **(c, d)**, and O*_x_* **(e, f)**.

Compared to the Base simulation, OH increases slightly by 1.1 % at the surface in the SAPRC simulation; with that, declines over the tropics (30° S–30° N) are compensated by enhancements over other regions (Fig. 10 and Table 2). The largest increases in OH concentrations are found over source regions dominated by anthropogenic emissions (i.e., the US, Europe, and Asia) and in subtropical continental regions with large biogenic aromatic emissions. In these locations, the peroxy radicals formed by aromatic oxidation react with NO and HO_2_, which can have a significant effect on the ambient ozone and NO*_x_* mixing ratios. This in turn influences OH, as the largest photochemical sources of OH in the model are the photolysis of O_3_ as well as the reaction of NO with HO_2_. Seasonally, a few surface locations see OH concentration increases of more than 10 % during April–August (not shown), including parts of the eastern US, central Europe, eastern Asia, and Japan.

**Figure 11.**
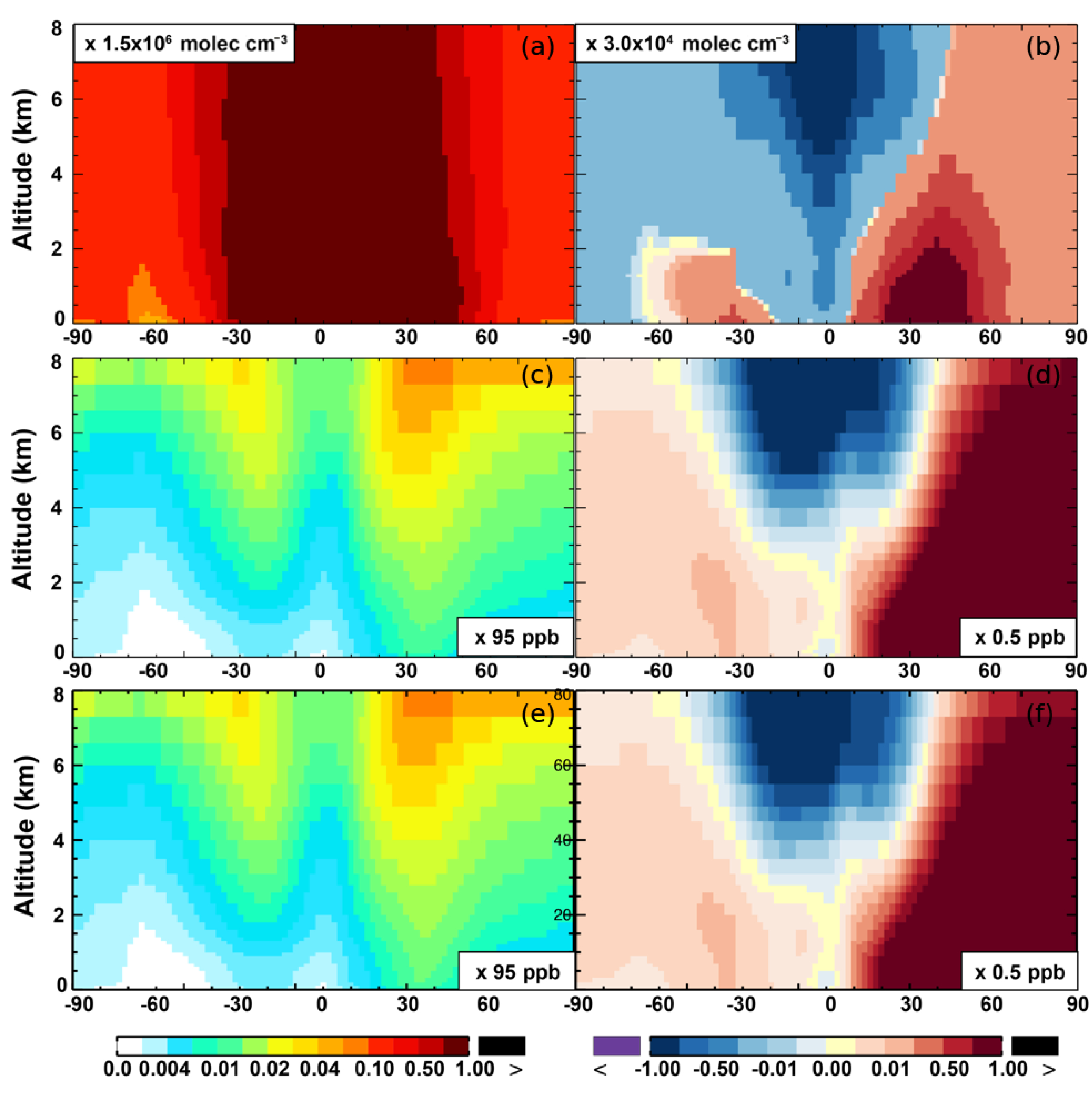
Same as Fig. 7 but for OH **(a, b)**, O_3_ **(c, d)**, and O*_x_* **(e, f)**.

**Figure 12.**
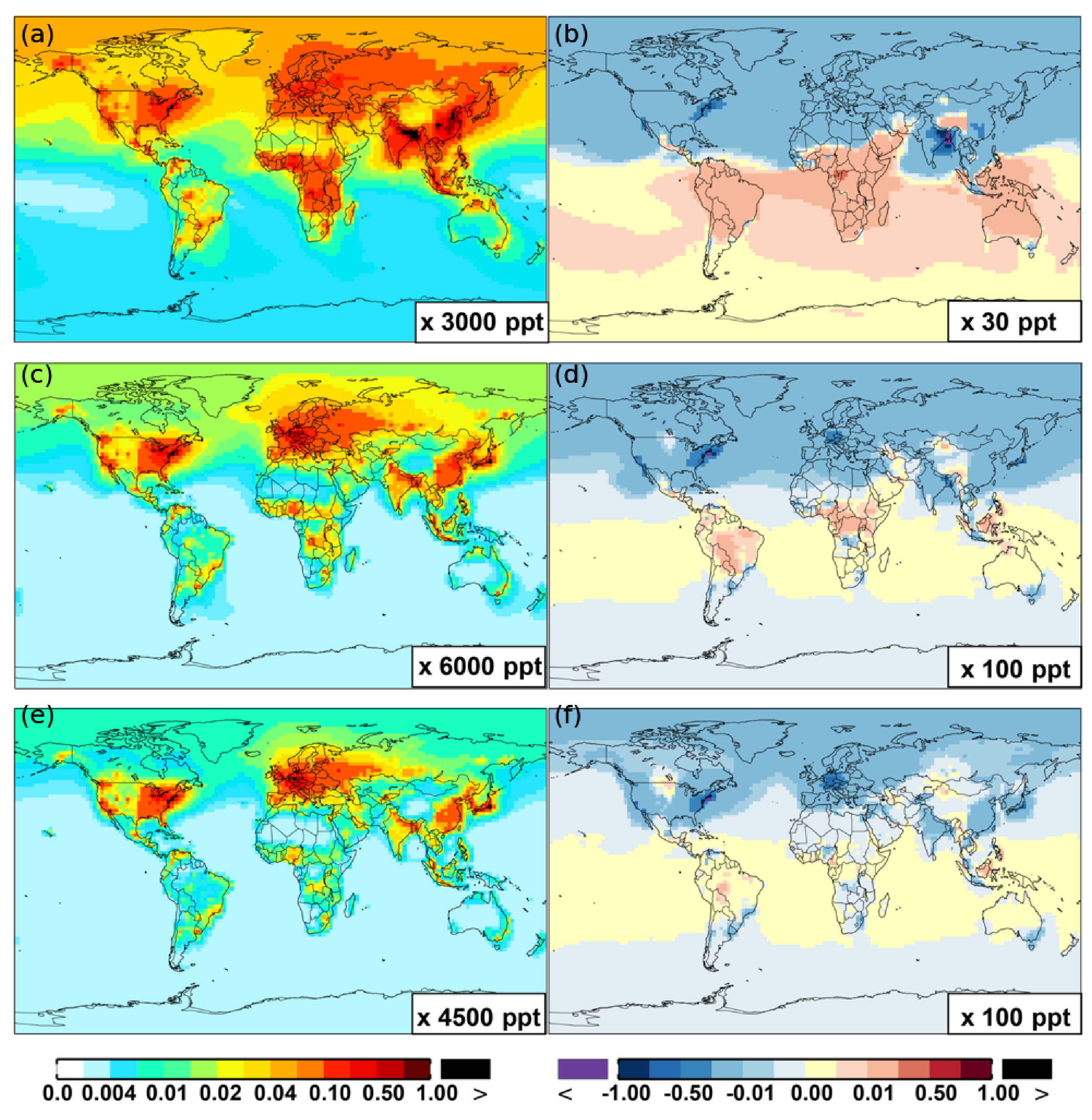
Same asFig. 6 but for benzene **(a, b)**, toluene **(c, d)**, and xylene **(e, f)**.

**Figure 13.**
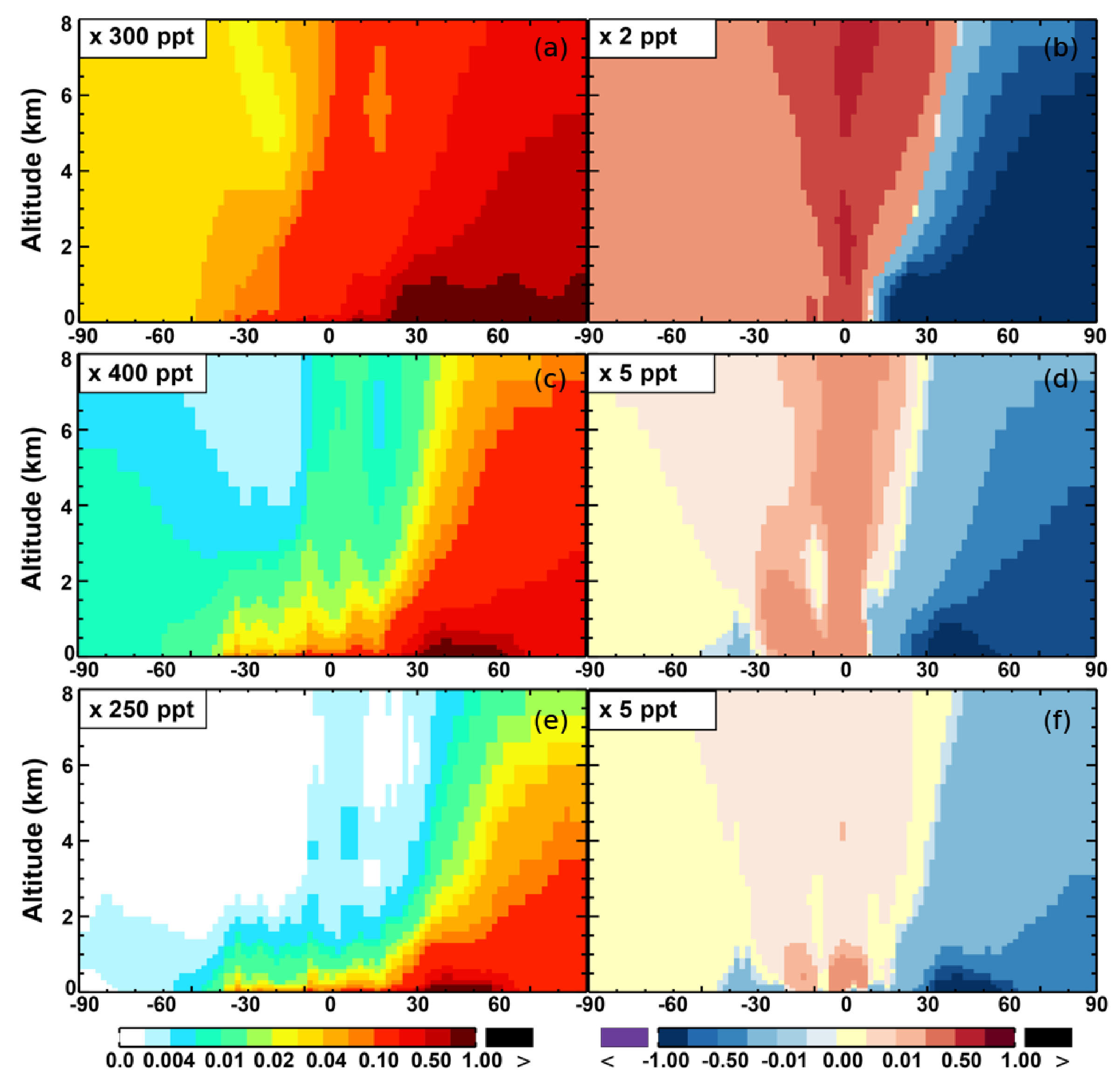
Same as Fig. 7 but for benzene **(a, b)**, toluene **(c, d)**, and xylene **(e, f)**.

The OH enhancement (0.2 %) is also seen in the free troposphere in the SAPRC simulation (Fig. 11 and Table 2). OH is increased in the troposphere of the Northern Hemisphere, in contrast to the decline in the troposphere of tropics and Southern Hemisphere (Fig. 11). These OH changes correspond to the hemispherically distinct changes in aromatics (benzene, toluene, and xylenes), which show a decrease in the Northern Hemisphere, an increase in the Southern Hemisphere (Figs. 12 and 13), and an increase in global mean (by 1 %) (Table 2). Despite the overall increase in tropospheric OH, CO is increased by ~ 1 % (Table 2) due to additional formation from aromatics oxidation.

Table 2 shows that, from Base to SAPRC, HO_2_ shows a significant increase at the global scale: 3.0 % at the surface and 1.3% in the troposphere, due to regeneration of HO_x_ from aromatics oxidation products. Correspondingly, the OH = HO_2_ ratio decreases slightly. These changes mean that, compared to the simplified aromatic chemistry in the standard model setup, the SAPRC mechanism is associated with higher OH (i.e., more chemically reactive troposphere) and even higher HO_2_.

### 5.3 Ozone

From Base to SAPRC, the global average surface ozone mixing ratio increases by less than 1 % (Table 2). This small difference is comparable to the result calculated by Cabrera-Perez et al. (2016) with the EMAC model, which is based on a reduced version of the aromatic chemistry from the Master Chemical Mechanism (MCMv3.2). Figure 10 shows that the 1 % increase in surface ozone occurs generally over the Northern Hemisphere. Similar to the changes in OH, the most notable ozone increase occurs in industrially polluted regions. These regions show significant local ozone photochemical formation in both the Base case and the SAPRC simulation. The updated aromatic chemistry increases ozone by up to 5 ppb in these regions. Increases of ozone are much smaller (less than 0.2 ppb) over the tropical oceans than in the continental areas. In contrast, ozone declines in regions of South America, central Africa, Australia, and Indonesia over the tropics (30° S–30° N). Changes elsewhere in the troposphere are similar in magnitude, as shown in Fig. 11.

Two general factors likely contribute to the ozone change from Base to SAPRC. In the SAPRC simulation, the addition of aromatic oxidation products (i.e., peroxy radicals) can contribute directly to ozone formation in NO*_x_*-rich source regions and also in the NO*_x_*-sensitive remote troposphere (i.e., from PAN to NO*_x_* and to ozone). The second factor is a change in the NO*_x_* spatial distribution, with an overall enhancement in average NO_2_ concentrations. The redistribution is mainly caused by enhanced transport of NO*_x_* to the remote troposphere (see Sect. 5.1). The enhanced NO*_x_* in the remote troposphere enhances the overall ozone formation because this process is more efficient in the remote regions (e.g., Liu et al., 1987). The increased ozone, NO_2_, and NO_x_ transport all lead to the aforementioned changes. This is described in detail in Sect. 5.4.

There are notable decreases (more than 5 %; Fig. 11) in simulated ozone and OH in the free troposphere (above 4 km) over the tropics (30° S–30° N). A similar decrease is found in modeled NO*_x_* (above 6 km; Fig. 9). These decreases are probably related to the upward transport of aromatics by tropical convection processes. The aromatics transported to the upper troposphere may cause net consumption of tropospheric OH and NO*_x_*, which can further reduce ozone production.

From Base to SAPRC, the modeled ozone concentrations are close to the WDCGG and EMEP network measurements (Table 3). For the WDCGG background sites, the annual and seasonal model biases are ∼ 10 % smaller in the SAPRC simulation compared to the Base case. For the EMEP stations, although the model results are not improved in summer and fall, the annual model bias is 25 % smaller (−2:8 ppb versus −3:5 ppb) in the SAPRC simulation.

### 5.4 Discussion of SAPRC aromatic–ozone chemistry

As discussed in Sect. 5.3, the increased O_3_ mixing ratios from Base to SAPRC are due to the direct impact of aromatic oxidation products (i.e., peroxy radicals) and to the effect of increased NO_2_ concentrations. The simulated odd oxygen family (O*_x_* = O_3_+ O(^1^D)+ O(^3^P)+NO_2_+ 2 × NO_3_+ 3×N_2_O_5_+HNO_3_+HNO_4_+PAN; Wu et al., 2007; Yan et al., 2016) formation increases by 1 %–10 %, both over the source regions and in the remote troposphere (Figs. 10 and 11). Although the percentage changes are similar, the driving factors over the source regions are different from the drivers in the remote troposphere.

Regions with large aromatics emissions show a significant increase of oxidation products from Base to SAPRC. The modeled ozone in these regions increases with increasing NO_2_ and its oxidation products. NO and NO_3_ are often lower in these regions in the SAPRC scenario because of their reactions with the aromatic–OH oxidation products to form NO_2_ and HO_2_. In remote regions and in the free troposphere, ozone production is also enhanced by both NO_2_ and HO_2_ increases in the SAPRC simulation, but the increase in ozone formation is mainly attributed to the increase in NO*_x_* mixing ratios.

NO*_x_* concentrations decrease in source regions and increase in the remote regions because of more efficient transport of PAN and its analogues (represented by PBZN here in SAPRC-11). From Base to SAPRC, modeled PAN has been enhanced on a global scale (Figs. 8 and 9) via reactions of aromatic–OH oxidation products with NO_2_ (equation of BR13 in Table S2). In the SAPRC-11 aromatics chemical scheme, the immediate precursor of PAN (peroxyacetyl radical) has five dominant photochemical precursors. They are acetone (CH_3_COCH_3_, model species: ACET), methacrolein (MACR), biacetyl (BACL), methyl glyoxal (MGLY), and other ketones (e.g., PROD2, AFG1). These compounds explain the increased rate of PAN formation. For example, the SAPRC simulation has increased the concentration of MGLY by a factor of 2. In addition, production of organic nitrates – PBZN (reactions of BR30 and BR31 in Table S2) and RNO_3_ (PO_36_) – in the model with SAPRC aromatics chemistry may also explain the increase in ambient NO*_x_* in the remote regions, due to the rerelease of NO*_x_* from organic nitrates (as opposed to removal by deposition). Due to such rerelease of NO*_x_* from PAN-like compounds and also transport of NO*_x_*, NO*_x_* increases by up to 5 % at the surface in most remote regions and by ∼ 1 % in the troposphere as a whole. This then leads to increased ozone due to the effectiveness of ozone formation in the free troposphere.

SAPRC is a highly efficient and compact chemical mechanism with the use of maximum ozone formation as a primary metric in the chamber experiment benchmark. The mechanism has been primarily used and evaluated in regional CTMs such as the Community Multi-scale Air Quality model (CMAQ) and Comprehensive Air Quality Model with Extensions (CAMx), at much finer resolution (i.e., a few kilometers). Our study has significant application to use it in a global model. Implementing SAPRC-11 aromatic chemistry would add ∼ 3 % more computational effort in terms of model simulation times.

SAPRC is based on lumped chemistry, which is partly optimized on empirical fitting to smog chamber experiments that are representative of 1-day photochemical smog episodes typical of, for example, Los Angeles and other US urban centers. However, SAPRC-11 gives better simulations of ozone formation in almost all conditions, except for higher (> 100 ppb) NO*_x_* experiments where O_3_ formation rates are consistently overpredicted (Carter and Heo, 2013). This overprediction can be corrected if the aromatics mechanism is parameterized to include a new NO*_x_* dependence on photoreactive product yields, but that parameterization is not incorporated in SAPRC-11 because it is inconsistent with available laboratory data.

Other options, such as the condensed MCM mechanism, which are based upon more fundamental laboratory and theoretical data and used for policy and scientific modeling of multi-day photochemical ozone formation, are experienced over Europe by Cabrera-Perez et al. (2016). Our results are consistent with the simulation of the EMAC model implemented with a reduced version of the MCM aromatic chemistry. Moreover, aromatic chemistry is still far from being completely understood. For example, Bloss et al. (2005) show that for alkyl substituted mono-aromatics, when compared to chamber experiments over a range of VOC/NO*_x_* conditions, the chemistry underpredicts the reactivity of the system but overpredicts the amount of O_3_ formation (model shows more NO-to-NO_2_ conversion than in the experiments).

## 6 Conclusions

A representation of tropospheric reactions for aromatic hydrocarbons in the SAPRC-11 mechanism has been added to GEOS-Chem to provide a more realistic representation of their atmospheric chemistry. The GEOS-Chem simulation with the SAPRC-11 aromatics mechanism has been evaluated against measurements from aircraft and surface campaigns. The comparison with observations shows reasonably good agreement for aromatics (benzene, toluene, and xylenes) and ozone. Model results for aromatics can reproduce the seasonal cycle, with a general underestimation over Europe for benzene and toluene, and an overestimation of xylenes; meanwhile, over the US, a positive model bias for benzene and toluene and a negative bias for C_8_ aromatics are found. From the Base to the SAPRC simulation, the model ozone bias is reduced by 10 % relative to WDCGG observations and by 25 % relative to EMEP observations.

The simplified aromatics chemistry in the Base simulation underpredicts NO and NO_3_ oxidation, and it does not represent ozone formed from aromatic–OH–NO*_x_* oxidation. Although the global average changes in simulated chemical species are relatively small (1 %–4 % from Base to SAPRC), on a regional scale, the differences can be much larger, especially over aromatics and NO_x_ source regions. From Base to SAPRC, NO_2_ is enhanced by up to 10 % over some highly polluted areas, while reductions are notable in other polluted areas. Although the simulated surface NO decreases by approximately 0.15 ppb (∼ 20 %) or more in the northern hemispheric source regions, including most of the US, Europe, and China, increases are found (∼ 0:1 ppb, up to 20 %) at locations downwind from these source regions. The total NO*_x_* mixing ratios decrease in source regions but increase in the remote free troposphere. This is mainly due to the addition of aromatic oxidation products in the model that lead to PAN, which facilitates the transport of nitrogen oxides to downwind locations remote from the sources. Finally, the updated aromatic chemistry in GEOS-Chem increases ozone concentrations, especially over industrialized regions (up to 5 ppb or more than 10 %). Ozone changes in the model are partly explained by the direct impact of increased aromatic oxidation products (i.e., peroxy radical) and partly by the effect of the altered spatial distribution of NO*_x_*. Overall, our results suggest that a better representation of aromatics chemistry is important to model the tropospheric oxidation capacity.

## Supplementary Material

SI

## Figures and Tables

**Table 1.** Summary of the statistical comparison between observed and simulated concentrations (ppt for aromatics, ppb for ozone). MMOD and MOBS represent the mean values for the SAPRC simulation and the observation, respectively. MRB is the relative bias of model results defined as (MMOD – MOBS)/MOBS. SMOD and SOBS are their standard deviations. TCOR and SCOR are the temporal and spatial correlations between model results and measurements.

Species	Network	No. of sites	Time resolution	MMOD (MRB)	MOBS	SMOD	SOBS	TCOR	SCOR

Benzene	CARIBIC	1241	Instantaneous	12.3 (−23 %)	16.0	4.2	15.8	–	0.31
	EEA	22	Annual mean	131.6 (−32 %)	194.0	32.1	118.4	–	0.49
	EMEP	14	Monthly	106.5 (−36 %)	166.4	38.7	71.7	0.77	0.44
	CALNEX	7708	Instantaneous	66.1 (15 %)	57.7	78.3	57.7	–	0.51
	KCMP	1	Hourly	99.9 (9 %)	91.5	92.6	56.7	0.65	–

Toluene	CARIBIC	789	Instantaneous	1.5 (−58 %)	3.6	0.7	7.5	–	0.36
	EEA	6	Annual mean	180.9 (−25 %)	240.3	66.8	59.4	–	0.41
	EMEP	12	Monthly	113.2 (−15 %)	133.1	47.3	66.2	0.81	0.47
	CALNEX	7708	Instantaneous	80.6 (10 %)	73.2	179.7	131.9	–	0.46
	KCMP	1	Hourly	121.2 (114 %)	56.7	191.4	54.7	0.51	–
Xylenes	EMEP	8	Monthly	78.4 (85 %)	42.3	34.5	41.9	0.78	0.48

C_8_ aromatics	CALNEX	7708	Instantaneous	28.8 (−41 %)	48.6	112.2	97.2	–	0.39
	KCMP	1	Hourly	88.9 (−2 %)	90.3	119.2	79.5	0.46	–

Ozone	WDCGG	64	Monthly	28.6 (−16 %)	34.1	12.8	14.2	0.68	0.54
	EMEP	130	Monthly	27.7 (−9 %)	30.6	13.2	10.3	0.76	0.52

**Table 2.** Annual and seasonal mean changes (%) in modeled surface as well as tropospheric concentrations from the Base to the SAPRC simulation. Also shown are the numbers for the Northern Hemisphere (NH) and Southern Hemisphere (SH).

Species	Annual		MAM		JJA		SON		DJF	
	Surface (NH, SH)	Trop (NH, SH)	Surface (NH, SH)	Trop (NH, SH)	Surface (NH, SH)	Trop (NH, SH)	Surface (NH, SH)	Trop (NH, SH)	Surface (NH, SH)	Trop (NH, SH)
NO	−0.2 % (−0.2 %, −1.4 %)	0.6 % (0.8 %, −0.2 %)	−0.4 % (−0.3 %, −1.7 %)	0.7 % (0.9 %, −0.3 %)	−1.3 % (−1.3 %, −1.2 %)	−0.1 % (−0.1 %, −0.1 %)	−1.5 % (−1.5 %, −1.3 %)	−0.5 % (−0.5 %, −0.3 %)	0.8 % (0.9 %, −1.6 %)	1.6 % (2.0 %, −0.3 %)
O_3_	0.9 % (1.2 %, 0.3 %)	0.4 % (0.6 %, −0.1 %)	1.1 % (1.6 %, 0.3 %)	0.5 % (0.8 %, −0.1 %)	0.6 % (0.9 %, 0.2 %)	0.3 % (0.5 %, −0.1 %)	0.8 % (1.1 %, 0.4 %)	0.4 % (0.6 %, −0.1 %)	1.0 % (1.3 %, 0.3 %)	0.4 % (0.6 %, −0.1 %)
CO	0.8 % (0.5 %, 1.3 %)	1.0 % (0.7 %, 1.4 %)	0.5 % (0.2 %, 1.1 %)	0.7 % (0.4 %, 1.3 %)	1.1 % (0.8 %, 1.4 %)	1.2 % (1.0 %, 1.5 %)	1.1 % (0.9 %, 1.5 %)	1.3 % (1.1 %, 1.6 %)	0.5 % (0.3 %, 1.0 %)	0.7 % (0.5 %, 1.2 %)
HNO_3_	1.1 % (1.3 %, −0.6 %)	0.3 % (0.7 %, −0.9 %)	1.2 % (1.3 %, −0.4 %)	0.4 % (0.7 %, −0.9 %)	0.7 % (0.9 %, −0.6 %)	−0.1 % (0.2 %, −1.0 %)	1.0 % (1.4 %, −0.7 %)	0.2 % (0.7 %, −1.0 %)	1.4 % (1.6 %, −0.7 %)	0.6 % (1.1 %, −0.8 %)
NO_2_	1.0 % (1.0 %, 0.2 %)	2.1 % (2.4 %, 0.7 %)	0.8 % (0.8 %, 0.3 %)	1.8 % (2.0 %, 0.8 %)	−0.2 % (−0.3 %, 0.1 %)	0.6 % (0.6 %, 0.8 %)	0.5 % (0.6 %, 0.2 %)	1.3 % (1.5 %, 0.5 %)	2.0 % (2.1 %, 0.2 %)	3.6 % (4.0 %, 0.5 %)
NO_3_	−0.9 % (−0.6 %, −2.7 %)	−4.1 % (−4.5 %, −3.5 %)	−1.5 % (−1.3 %, −2.7 %)	−5.6 % (−7.0 %, −3.0 %)	−0.9 % (−0.5 %, −2.5 %)	−3.7 % (−4.3 %, −3.0 %)	−0.5 % (−0.1 %, −2.6 %)	−3.4 % (−3.4 %, −3.6 %)	−0.8 % (−0.5 %, −3.6 %)	−4.1 % (−4.2 %, −4.5 %)
BENZ	−0.5 % (−0.6 %, 0.6 %)	−0.4 % (−0.6 %, 1.4 %)	−0.9 % (−1.0 %, 0.7 %)	−1.0 % (−1.1 %, 1.7 %)	0.1 % (−0.1 %, 0.5 %)	0.7 % (0.5 %, 1.0 %)	−0.1 % (−0.2 %, 0.8 %)	0.2 % (−0.1 %, 1.6 %)	−0.6 % (−0.6 %, 0.9 %)	−0.6 % (−0.7 %, 2.0 %)
TOLU	−1.2 % (−1.3 %, 0.1 %)	−1.9 % (−2.0 %, 0.4 %)	−1.5 % (−1.6 %, 0.3 %)	−2.8 % (−3.0 %, 0.8 %)	−0.8 % (−1.0 %, −0.2 %)	−0.9 % (−1.2 %, −0.1 %)	−1.0 % (−1.1 %, 0.2 %)	−1.5 % (−1.6 %, 0.6 %)	−1.3 % (−1.3 %, 0.4 %)	−1.9 % (−2.0 %, 1.3 %)
XYLE	−1.4 % (−1.5 %, −0.3 %)	−2.3 % (−2.3 %, −0.2 %)	−1.2 % (−1.2 %, −0.2 %)	−2.1 % (−2.2 %, 0.3 %)	−1.2 % (−1.3 %, −0.6 %)	−1.5 % (−1.6 %, −0.9 %)	−1.6 % (−1.7 %, −0.1 %)	−2.3 % (−2.4 %, 0.2 %)	−1.5 % (−1.5 %, −0.1 %)	−2.4 % (−2.4 %, 0.5 %)
OH	1.1 % (1.6 %, 0.3 %)	0.2 % (0.6 %, −0.3 %)	1.4 % (1.9 %, 0.3 %)	0.4 % (0.8 %, −0.4 %)	1.2 % (1.3 %, 0.5 %)	0.3 % (0.5 %, −0.2 %)	0.9 % (1.5 %, 0.3 %)	0.1 % (0.4 %, −0.4 %)	1.0 % (2.1 %, 0.2 %)	0.1 % (0.9 %, −0.3 %)
HO_2_	3.0 % (3.2 %, 2.8 %)	1.3 % (1.4 %, 1.2 %)	2.9 % (2.8 %, 3.1 %)	1.4 % (1.5 %, 1.2 %)	3.3 % (3.2 %, 3.6 %)	1.3 % (1.2 %, 1.6 %)	3.1 % (3.4 %, 2.8 %)	1.3 % (1.5 %, 1.2 %)	2.8 % (3.7 %, 2.2 %)	1.2 % (1.9 %, 0.9 %)
OH/HO_2_	−1.4 % (−1.0 %, −1.7 %)	−0.9 % (−0.7 %, −1.3 %)	−1.2 % (−1.1 %, −1.9 %)	−0.8 % (−0.5 %, −1.4 %)	−1.6 % (−1.1 %, −2.0 %)	−1.0 % (−0.7 %, −1.6 %)	−1.4 % (−0.9 %, −1.9 %)	−1.0 % (−0.8 %, −1.4 %)	−1.1 % (−0.5 %, −2.1 %)	−0.8 % (−0.6 %, −1.3 %)

**Table 3.** Annual and seasonal mean model ozone biases for the Base and the SAPRC cases, compared to measurements from WDCGG and EMEP.

Species (ppb)	Annual		MAM		JJA		SON		DJF	
	Base	SAPRC	Base	SAPRC	Base	SAPRC	Base	SAPRC	Base	SAPRC

WDCGG	−6.0	−5.4	−9.0	−8.4	−0.4	0.1	−2.5	−2.1	−11.9	−11.5
EMEP	−3.5	−2.8	−5.5	−4.7	4.5	5.2	0.3	0.8	−13.1	−12.8

## Data Availability

The aircraft and surface data used in this paper are already publicly available. Airborne observations of aromatics are from CALNEX:
pre-generated data files, available at https://www.esrl.noaa.gov/csd/projects/calnex, last access: 28 December 2018; European Monitoring and Evaluation Programme (EMEP): pre-generated data files, available at http://www.nilu.no/projects/ccc/emepdata.html, last access: 28 December 2018; EEA: pre-generated data files, available at http://www.eea.europa.eu/data-and-maps/data/airbase-the-european-air-quality-database-8, last access: 28 December 2018; KCMP: pre-generated data files, available at https://atmoschem.umn.edu/data, last access: 28 December 2018; and World Data Centre for Greenhouse Gases (WDCGG): pre-generated data files, available at http://ds.data.jma.go.jp/gmd/wdcgg/cgi-bin/wdcgg/catalogue.cgi, last access: 28 December 2018.

## Code availability.

The GEOS-Chem code of version 9–02 used to generate this paper and the model results are available upon request. We are submitting the code for inclusion into the standard model. The revised aromatics chemistry will be incorporated in the current version (12.0.0) and the later versions.
