# Supplementary material for: Global tropospheric effects of aromatic chemistry with the SAPRC-11 mechanism implemented in GEOS-Chem version 9-02
Source: Geosci Model Dev. Author manuscript; Available in PMC 2021 Feb 19. (PMC7894209; doi:10.5194/gmd-12-111-2019)
Supplement: SI [file NIHMS1669562-supplement-SI.pdf]

Supplement of Geosci. Model Dev., 12, 111–130, 2019
https://doi.org/10.5194/gmd-12-111-2019-supplement
© Author(s) 2019. This work is distributed under
the Creative Commons Attribution 4.0 License.

Geoscientific
Model Development
Open Access

Supplement of

Global tropospheric effects of aromatic chemistry with the SAPRC-11 mechanism implemented in GEOS-Chem version 9-02

Yingying Yan et al.

Correspondence to: Jintai Lin (linjt@pku.edu.cn)

The copyright of individual parts of the supplement might differ from the CC BY 4.0 License.

Table S1. List of relevant model species from the SAPRC-11 mechanism.

Type and Name	Description
BENZ (Existing)	Benzene
TOLU (Existing)	Toluene
XYLE (Existing)	Xylene
Active Radical Species and Operators	
MO2 (Existing)	Methyl Peroxy Radicals
RO2C (New)	Peroxy Radical Operator representing NO to NO2 and NO3 to NO2 conversions, and the effects of peroxy radical reactions on acyl peroxy and other peroxy radicals.
RO2XC (New)	Peroxy Radical Operator representing NO consumption (used in conjunction with organic nitrate formation), and the effects of peroxy radical reactions on NO3, acyl peroxy radicals, and other peroxy radicals.
MCO3 (Existing)	Acetyl Peroxy Radicals
RCO3 (Existing)	Peroxy Propionyl and higher peroxy acyl Radicals
BZCO3 (New)	Peroxyacyl radical formed from Aromatic Aldehydes
MACO3 (New)	Peroxyacyl radicals formed from methacrolein and other acroleins.
Steady State Radical Species	
BZO (New)	Phenoxy Radicals
HCOCO3 (New)	HC(O)C(O)OO Radicals
PBZN (New)	PAN analogues formed from Aromatic Aldehydes
Explicit and Lumped Molecule Reactive Organic Product Species	
CH2O (Existing)	Formaldehyde
ALD2 (Existing)	Acetaldehyde
RCHO (Existing)	Lumped C3+ Aldehydes (mechanism based on propionaldehyde)
ACET (Existing)	Acetone
MOH (Existing)	Methanol
HCOOH (Existing)	Formic Acid
MAP (Existing)	Acetic Acid. Also used for peroxyacetic acid.
RCOOH (Existing)	Higher organic acids and peroxy acids (mechanism based on propionic acid)
MP (Existing)	Methyl Hydroperoxide
ROOH (New)	Lumped organic hydroperoxides with 2-4 carbons. Mechanism based on that estimated for npropyl hydroperoxide.
R6OOH (New)	Lumped organic hydroperoxides with 5 or more carbons, and organic hydroperoxides formed from aromatics that do not participate in SOA formation. Mechanism based on that estimated for 3-hexyl hydroperoxide.
RAOOH (New)	Organic hydroperoxides formed from aromatic hydrocarbons that condense to form SOA (see Carter et al, 2012 for a complete listing of the mechanism and model species used for modeling aromatic SOA formation.)
GLYX (Existing)	Glyoxal
BACL (New)	Biacetyl
PHEN (New)	Phenol
CRES (New)	Cresols
XYNL (New)	Xylenols and higher alkylphenols

CATL (New)	Catechols
NPHE (New)	Nitrophenols
BALD (New)	Aromatic aldehydes (e.g., benzaldehyde)
MACR (Existing)	Methacrolein
MVK (New)	Methyl Vinyl Ketone
IPRD (New)	Lumped isoprene product species
Aromatic unsaturated ring fragmentation products	
AFG1 (New)	Monounsaturated dialdehydes or aldehyde-ketones formed from aromatics. - Most photoreactive
AFG2 (New)	Monounsaturated dialdehydes or aldehyde-ketones formed from aromatics. - Least photoreactive
AFG3 (New)	Diunsaturatred dicarbonyl aromatic fragmentation products that are assumed not to photolyze rapidly
Lumped Parameter Products	
PROD2 (New)	Ketones and other non-aldehyde oxygenated products which react with OH radicals faster than 5 x 10-12 cm3 molec-2 sec-1.
RNO3 (New)	Lumped Organic Nitrates
Steady state operators used to represent radical or product formation in peroxy radical reactions.	
xHO2 (New)	Formation of HO2 from alkoxy radicals formed in peroxy radical reactions with NO and NO3 (100% yields) and RO2 (50% yields)
xOH (New)	As above, but for OH
xNO2 (New)	As above, but for NO2
xMO2 (New)	As above, but for MO2
xMCO3 (New)	As above, but for MCO3
xRCO3 (New)	As above, but for RCO3
xMACO3 (New)	As above, but for MACO3
xCO (New)	As above, but for CO
xCH2O (New)	As above, but for CH2O
xALD2 (New)	As above, but for ALD2
xRCHO (New)	As above, but for RCHO
xACET (New)	As above, but for ACET
xMEK (New)	As above, but for MEK
xPROD2 (New)	As above, but for PROD2
xBALD (New)	As above, but for BALD
xGLYX (New)	As above, but for GLYX
xMGLY (New)	As above, but for MGLY
xBACL (New)	As above, but for BACL
xAFG1 (New)	As above, but for AFG1
xAFG2 (New)	As above, but for AFG2
xAFG4 (New)	As above, but for AFG4
xMACR (New)	As above, but for MACR
xMVK (New)	As above, but for MVK
xIPRD (New)	As above, but for IPRD
xRNO3 (New)	As above, but for RNO3
zRNO3 (New)	Formation of RNO3 in the RO2 + NO, reaction, or formation of corresponding non-nitrate products (represented by PROD2) formed from alkoxy radicals formed in RO2 + NO3 and (in 50% yields) RO2 + RO2 reactions.

yROOH (New)	Formation of ROOH following RO2 + HO2 reactions, or formation of H-shift disproportionation products (represented by MEK) in the RO2 + RCO3 and (in 50% yields) RO2 + RO2 reactions.
yR6OOH (New)	As above, but the RO2 + HO2 product is represented by R6OOH and the H-shift products are represented by PROD2.
yRAOOH (New)	Like yROOH or yR6OOH but for RAOOH
Non-Reacting Species	
XC (New)	Lost Carbon or carbon in unreactive products
XN (New)	Lost Nitrogen or nitrogen in unreactive products

Table S2. Listing of relevant reactions and rate parameters from the base SAPRC-11 mechanism. "#" refers to zero.

Label	Label Reaction and Products	Rate Parameters			
		k(300)	A	Ea	B
Methyl peroxy and methoxy reactions					
BR01(Updated)	MO2 + NO = NO2 + CH2O + HO2	7.64e-12	2.30e-12	-0.72	
BR02(Updated)	MO2 + HO2 = MP + O2	4.65e-12	3.46e-13	-1.55	0.36
BR03(Updated)	MO2 + HO2 = CH2O + O2 + H2O	4.50e-13	3.34e-14	-1.55	-3.53
BR04 (New)	MO2 + NO3 = CH2O + HO2 + NO2	1.30e-12			
BR05(Updated)	MO2 + MO2 = MOH + CH2O + O2	2.16e-13	6. 39e-14	-0.73	-1.80
BR06(Updated)	MO2 + MO2 = #2 {CH2O + HO2}	1.31e-13	7.40e-13	1.03	
Active Peroxy Racical Operators					
BR07 (New)	RO2C + NO = NO2	9.23e-12	2.60e-12	-0.76	
BR08 (New)	RO2C + NO3 = NO2	2.30e-12			
BR09 (New)	RO2C + MO2 = #.5 HO2 + #.75 CH2O + #.25 MOH	2.00e-13			
BR10 (New)	RO2XC + NO = XN	Same k as rxn BR07			
BR11 (New)	RO2XC + NO3 = NO2	Same k as rxn BR08			
BR12 (New)	RO2XC + MO2 = #.5 HO2 + #.75 CH2O + #.25 MOH	Same k as rxn BR09			
Reactions of Acyl Peroxy Radicals, PAN, and PAN analogues					
BR13(Updated)	MCO3 + NO2 = PAN	9.37e-12			
BR14(Updated)	MCO3 + NO = MO2 + CO2 + NO2	1.97e-11	7.50e-12	-0.58	
BR15(Updated)	MCO3 + HO2 = #.44 {OH + MO2 + CO2} + #.41 MAP + #.15 {O3 + MAP}	1.36e-11	5.20e-13	-1.95	
BR16 (New)	MCO3 + NO3 = MO2 + CO2 + NO2 + O2	Same k as rxn BR08			
BR17(Updated)	MCO3 + MO2 = #.1 {MAP + CH2O + O2} + #.9 {CH2O + HO2 + MO2 + CO2}	1.06e-11	2.00e-12	-0.99	
BR18 (New)	MCO3 + RO2C = MO2 + CO2	1.56e-11	4.40e-13	-2.13	
BR19 (New)	MCO3 + RO2XC = MO2 + CO2	Same k as rxn BR18			
BR20(Updated)	MCO3 + MCO3 = #2 {MO2 + CO2} + O2	1.54e-11	2.90e-12	-0.99	
BR21 (New)	RCO3 + NO = NO2 + RO2C + xHO2 + yROOH + xALD2 + CO2	2.08e-11	6.70e-12	-0.68	
BR22 (New)	RCO3 + HO2 = #.44 {OH + RO2C + xHO2 + xALD2 + yROOH + CO2} + #.41 RCOOH + #.15 {O3 + RCOOH}	Same k as rxn BR15			
BR23 (New)	RCO3 + NO3 = NO2 + RO2C + xHO2 + yROOH + xALD2 + CO2 + O2	Same k as rxn BR08			
BR24 (New)	RCO3 + MO2 = CH2O + HO2 + RO2C + xHO2 + xALD2 + yROOH + CO2	Same k as rxn BR17			
BR25 (New)	RCO3 + RO2C = RO2C + xHO2 + xALD2 + yROOH + CO2	Same k as rxn BR18			
BR26 (New)	RCO3 + RO2XC = RO2C + xHO2 + xALD2 + yROOH + CO2	Same k as rxn BR18			
BR27 (New)	RCO3 + MCO3 = #2 CO2 + MO2 + RO2C + xHO2 + yROOH + xALD2 + O2	Same k as rxn BR20			
BR28 (New)	RCO3 + RCO3 = #2 {RO2C + xHO2 + xALD2 + yROOH+ CO2}	Same k as rxn BR20			
BR29 (New)	BZCO3 + NO2 = PBZN	1.37e-11			
BR30 (New)	PBZN = BZCO3 + NO2	4.27e-4	7.90e+16	27.82	
BR31 (New)	PBZN + HV = #.6 {BZCO3 + NO2} + #.4 {CO2 + BZO + RO2C + NO3}	Phot Set= PAN			
BR32 (New)	BZCO3 + NO = NO2 + CO2 + BZO + RO2C	Same k as rxn BR21			
BR33 (New)	BZCO3 + HO2 = #.44 {OH + BZO + RO2C +	Same k as rxn BR15			

	CO2} + #.41 RCOOH + #.15 {O3 + RCOOH} + #2.24 XC				
BR34 (New)	BZCO3 + NO3 = NO2 + CO2 + BZO + RO2C + O2	Same k as rxn BR08			
BR35 (New)	BZCO3 + MO2 = CH2O + HO2 + RO2C + BZO + CO2	Same k as rxn BR17			
BR36 (New)	BZCO3 + RO2C = RO2C + BZO + CO2	Same k as rxn BR18			
BR37 (New)	BZCO3 + RO2XC = RO2C + BZO + CO2	Same k as rxn BR18			
BR38 (New)	BZCO3 + MCO3 = #2 CO2 + MO2 + BZO + RO2C	Same k as rxn BR20			
BR39 (New)	BZCO3 + RCO3 = #2 CO2 + RO2C + xHO2 + yROOH + xALD2 + BZO + RO2C	Same k as rxn BR20			
BR40 (New)	BZCO3 + BZCO3 = #2 {BZO + RO2C + CO2}	Same k as rxn BR20			
BR42 (New)	MACO3 + NO = NO2 + CO2 + CH2O + MCO3	Same k as rxn BR21			
BR43 (New)	MACO3 + HO2 = #.44 {OH + CH2O + MCO3 + CO2} + #.41 RCOOH + #.15 {O3 + RCOOH} + #.56 XC	Same k as rxn BR15			
BR44 (New)	MACO3 + NO3 = NO2 + CO2 + CH2O + MCO3 + O2	Same k as rxn BR08			
BR45 (New)	MACO3 + MO2 = #2 CH2O + HO2 + CO2 + MCO3	Same k as rxn BR17			
BR46 (New)	MACO3 + RO2C = CO2 + CH2O + MCO3	Same k as rxn BR18			
BR47 (New)	MACO3 + RO2XC = CO2 + CH2O + MCO3	Same k as rxn BR18			
BR48 (New)	MACO3 + MCO3 = #2 CO2 + MO2 + CH2O + MCO3 + O2	Same k as rxn BR20			
BR49 (New)	MACO3 + RCO3 = CH2O + MCO3 + RO2C + xHO2 + yROOH + xALD2 + #2 CO2	Same k as rxn BR20			
BR50 (New)	MACO3 + BZCO3 = CH2O + MCO3 + BZO + RO2C + #2 CO2	Same k as rxn BR20			
BR51 (New)	MACO3 + MACO3 = #2 {CH2O + MCO3 + CO2}	Same k as rxn BR20			
Other Organic Radical Species					
BR52 (New)	BZO + NO2 = NPHE	3.79e-11	2.30e-11	-0.30	
BR53 (New)	BZO + HO2 = CRES + #-1 XC	7.63e-12	3.80e-13	-1.79	
BR54 (New)	BZO = CRES + RO2C + xHO2 + #-1 XC	1.00e-3			
Explicit and Lumped Molecule Organic Products					
BP01 (Updated)	ALD2 + OH = MCO3 + H2O	1.49e-11	4.40e-12	-0.73	
BP02 (Updated)	ALD2 + HV = CO + HO2 + MO2	Phot Set= ALD2_R			
BP03 (Updated)	ALD2 + NO3 = HNO3 + MCO3	2.84e-15	1.40e-12	3.70	
BP04 (Updated)	RCHO + OH = #.965 RCO3 + #.035 {RO2C + xHO2 + xCO + xALD2 + yROOH}	1.97e-11	5.10e-12	-0.80	
BP05 (Updated)	RCHO + HV = RO2C + xHO2 + yROOH + xALD2 + CO + HO2	Phot Set= C2CHO			
BP06 (Updated)	RCHO + NO3 = HNO3 + RCO3	6.74e-15	1.40e-12	3.18	
BP07 (Updated)	ACET + OH = RO2C + xMCO3 + xCH2O + yROOH	1.91e-13	4.56e-14	-0.85	3.65
BP08 (Updated)	ACET + HV = #.62 MCO3 + #1.38 MO2 + #.38 CO	Phot Set= ACET-06, qy= 0.5			
BP09 (Updated)	MOH + OH = CH2O + HO2	9.02e-13	2.85e-12	0.69	
BP10 (Updated)	MAP + OH = #.509 MO2 + #.491 RO2C + #.509 CO2 + #.491 xHO2 + #.491 xMGLY + #.491 yROOH + #-0.491 XC	7.26e-13	4.20e-14	-1.70	
BP11 (Updated)	MP + OH = H2O + #.3 {CH2O + OH} + #.7 MO2	7.40e-12	3.80e-12	-0.40	
BP12 (Updated)	MP + HV = CH2O + HO2 + OH	Phot Set= MP			

BP13 (Updated)	MACR + OH = #.5 MACO3 + #.5 {RO2C + xHO2} + #.416 xCO + #.084 xCH2O + #.416 xMEK + #.084 xMGLY + #.5 yROOH + #-0.416 XC	2.84e-11	8.00e-12	-0.76	
BP14 (Updated)	MACR + O3 = #.208 OH + #.108 HO2 + #.1 RO2C + #.45 CO + #.117 CO2 + #.1 CH2O + #.9 MGLY + #.333 HCOOH + #.1 xRCO3 + #.1 xCH2O + #.1 yROOH + #-0.1 XC	1.28e-18	1.40e-15	4.17	
BP15 (Updated)	MACR + NO3 = #.5 {MACO3 + RO2C + HNO3 + xHO2 + xCO} + #.5 yROOH + #1.5 XC + #.5 XN	3.54e-15	1.50e-12	3.61	
BP16 (Updated)	MACR + HV = #.33 OH + #.67 HO2 + #.34 MCO3 + #.33 MACO3 + #.33 RO2C + #.67 CO + #.34 CH2O + #.33 xMCO3 + #.33 xCH2O + #.33 yROOH	Phot Set= MACR-06			
BP17 (New)	MVK + OH = #.975 RO2C + #.025 {RO2XC + zRNO3} + #.3 xHO2 + #.675 xMCO3 + #.3 xCH2O + #.675 xRCHO + #.3 xMGLY + yROOH + #-0.725 XC	1.99e-11	2.60e-12	-1.21	
BP18 (New)	MVK + O3 = #.164 OH + #.064 HO2 + #.05 {RO2C + xHO2} + #.475 CO + #.124 CO2 + #.05 CH2O + #.95 MGLY + #.351 HCOOH + #.05 xRCO3 + #.05 xCH2O + #.05 yROOH + #-0.05 XC	5.36e-18	8.50e-16	3.02	
BP19 (New)	MVK + NO3 = #4 XC + XN	Slow			
BP20 (New)	MVK + HV = #.4 MO2 + #.6 CO + #.6 PROD2 + #.4 MACO3 + #-2.2 XC	Phot Set= MVK-06			
BP21 (New)	IPRD + OH = #.289 MACO3 + #.67 {RO2C + xHO2} + #.041 {RO2XC + zRNO3} + #.336 xCO + #.055 xCH2O + #.129 xALD2 + #.013 xRCHO + #.15 xMEK + #.332 xPROD2 + #.15 xGLYX + #.174 xMGLY + #-0.504 XC + #.711 yR6OOH	6.19e-11			
BP22 (New)	IPRD + O3 = #.285 OH + #.4 HO2 + #.048 {RO2C + xRCO3} + #.498 CO + #.14 CO2 + #.124 CH2O + #.21 MEK + #.023 GLYX + #.742 MGLY + #.1 HCOOH + #.372 RCOOH + #.047 xALD2 + #.001 xCH2O + #.048 yR6OOH + #-.329 XC	4.18e-18			
BP23 (New)	IPRD + NO3 = #.15 {MACO3 + HNO3} + #.799 {RO2C + xHO2} + #.051 {RO2XC + zRNO3} + #.572 xCO + #.227 xCH2O + #.218 xRCHO + #.008 xMGLY + #.572 xRNO3 + #.85 yR6OOH + #.278 XN + #-.815 XC	1.00e-13			
BP24 (New)	IPRD + HV = #1.233 HO2 + #.467 MCO3 + #.3 RCO3 + #1.233 CO + #.3 CH2O + #.467 ALD2 + #.233 MEK + #-.233 XC	Phot Set= MACR-06			
Lumped Parameter Organic Products					
BP25 (New)	PROD2 + OH = #.472 HO2 + #.379 xHO2 + #.029 xMCO3 + #.049 xRCO3 + #.473 RO2C + #.071 RO2XC + #.071 zRNO3 + #.002 CH2O + #.211 xCH2O + #.001 ALD2 + #.083 xALD2 + #.143 RCHO + #.402 xRCHO + #.115 xMEK + #.329 PROD2 + #.007 xPROD2 + #.528 yR6OOH + #.877 XC	1.55e-11			
BP26 (New)	PROD2 + HV = #.913 xHO2 + #.4 MCO3 + #.6 RCO3 + #1.59 RO2C + #.087 RO2XC +	Phot Set= MEK-06, qy= 4.86e-3			

	#.087 zRNO3 + #.303 xCH2O + #.163 xALD2 + #.78 xRCHO + yR6OOH + #- .091 XC				
Aromatic Products					
BP27 (Updated)	GLYX + HV = #2 {CO + HO2}	Phot Set= GLYX-07R			
BP28 (Updated)	GLYX + HV = CH2O + CO	Phot Set= GLYX-07M			
BP29 (Updated)	GLYX + OH = #.7 HO2 + #1.4 CO + #.3 HCOCO3	9.63e-12	3.10e-12	-0.68	
BP30 (Updated)	GLYX + NO3 = HNO3 + #.7 HO2 + #1.4 CO + #.3 HCOCO3	1.02e-15	2.80e-12	4.72	
BP31 (New)	HCOCO3 + NO = HO2 + CO + CO2 + NO2	Same k as rxn BR21			
BP32 (New)	HCOCO3 + NO2 = HO2 + CO + CO2 + NO3	1.21e-11	1.21e-11	0.0	-1.07
BP33 (New)	HCOCO3 + HO2 = #.44 {OH + HO2 + CO + CO2} + #.56 GLYX + #.15 O3	Same k as rxn BR15			
BP34 (New)	BACL + HV = #2 MCO3	Phot Set= BACL-07			
BP35 (New)	NPHE + OH = BZO + XN	3.50e-12			
BP36 (New)	NPHE + HV = HONO + #6 XC	Phot Set= NO2-06, qy= 1.5e-3			
BP37 (New)	NPHE + HV = #6 XC + XN	Phot Set= NO2-06, qy= 1.5e-2			
BP38 (New)	BALD + OH = BZCO3	1.20e-11			
BP39 (New)	BALD + HV = #7 XC	Phot Set= BALD-06, qy= 0.06			
BP40 (New)	BALD + NO3 = HNO3 + BZCO3	2.73e-15	1.34e-12	3.70	
BP41 (New)	PHEN + OH = #.7 HO2 + #.1 BZO + #.095 xHO2 + #.105 OH + #.095 RO2C + #.7 CATL + #.105 AFG3 + #.048 xAFG1 + #.048 xAFG2 + #.095 xGLYX + #.095 yRAOOH	2.74e-11	4.70e-13	-2.42	
BP42 (New)	PHEN + NO3 = #.1 HNO3 + #.9 XN + #.7 HO2 + #.1 BZO + #.095 xHO2 + #.105 OH + #.095 RO2C + #.7 CATL + #.105 AFG3 + #.048 xAFG1 + #.048 xAFG2 + #.095 xGLYX + #.095 yRAOOH	3.80e-12			
BP43 (New)	CRES + OH = #.7 HO2 + #.1 BZO + #.17 xHO2 + #.03 OH + #.17 RO2C + #.7 CATL + #.03 AFG3 + #.085 xAFG1 + #.085 xAFG2 + #.085 xGLYX + #.085 xMGLY + #.17 yRAOOH	4.06e-11	1.60e-12	-1.93	
BP44 (New)	CRES + NO3 = #.1 HNO3 + #.9 XN + #.7 HO2 + #.1 BZO + #.17 xHO2 + #.03 OH + #.17 RO2C + #.7 CATL + #.03 AFG3 + #.085 xAFG1 + #.085 xAFG2 + #.085 xGLYX + #.085 xMGLY + #.170 yRAOOH	1.40e-11			
BP45 (New)	XYNL + OH = #.7 HO2 + #.07 BZO + #.23 xHO2 + #.23 RO2C + #.7 CATL + #.115 xAFG1 + #.115 xAFG2 + #.115 xGLYX + #.115 xMGLY + #.23 yRAOOH	7.38e-11			
BP46 (New)	XYNL + NO3 = #.07 HNO3 + #.93 XN + #.7 HO2 + #.07 BZO + #.23 xHO2 + #.23 RO2C + #.7 CATL + #.115 xAFG1 + #.115 xAFG2 + #.115 xGLYX + #.115 xMGLY + #.23 yRAOOH	3.06e-11			
BP47 (New)	CATL + OH = #.4 HO2 + #.2 BZO + #.2 xHO2 + #.2 OH + #.2 RO2C + #.2 AFG3 + #.1 xAFG1 + #.1 xAFG2 + #.1 xGLYX + #.1 xMGLY + #.33 CNDPP + #.2 yRAOOH	2.00e-10			
BP48 (New)	CATL + NO3 = #.2 HNO3 + #.8 XN + #.4 HO2 + #.2 BZO + #.2 xHO2 + #.2 OH + #.2	1.70e-10			

	RO2C + #.2 AFG3 + #.1 xAFG1 + #.1 xAFG2 + #.1 xGLYX + #.1 xMGLY + #.2 yRAOOH				
BP49 (New)	AFG1 + OH = #.217 MACO3 + #.723 RO2C + #.060 {RO2XC + zRNO3} + #.521 xHO2 + #.201 xMCO3 + #.334 xCO + #.407 xRCHO + #.129 xMEK + #.107 xGLYX + #.267 xMGLY + #.783 yR6OOH + #.284 XC	7.40e-11			
BP50 (New)	AFG1 + HV = #1.023 HO2 + #.173 MO2 + #.305 MCO3 + #.500 MACO3 + #.695 CO + #.195 GLYX + #.305 MGLY + #.217 XC	Phot Set= AFG1			
BP51 (New)	AFG2 + OH = #.217 MACO3 + #.723 RO2C + #.060 {RO2XC + zRNO3} + #.521 xHO2 + #.201 xMCO3 + #.334 xCO + #.407 xRCHO + #.129 xMEK + #.107 xGLYX + #.267 xMGLY + #.783 yR6OOH + #.284 XC	7.40e-11			
BP52 (New)	AFG2 + HV = PROD2 + #-1 XC	Phot Set= AFG1			
BP53 (New)	AFG3 + OH = #.206 MACO3 + #.733 RO2C + #.117 {RO2XC + zRNO3} + #.561 xHO2 + #.117 xMCO3 + #.114 xCO + #.274 xGLYX + #.153 xMGLY + #.019 xBACL + #.195 xAFG1 + #.195 xAFG2 + #.231 xIPRD + #.794 yR6OOH + #.938 XC	9.35e-11			
BP54 (New)	AFG3 + O3 = #.471 OH + #.554 HO2 + #.013 MCO3 + #.258 RO2C + #.007 {RO2XC + zRNO3} + #.580 CO + #.190 CO2 + #.366 GLYX + #.184 MGLY + #.350 AFG1 + #.350 AFG2 + #.139 AFG3 + #.003 MACR + #.004 MVK + #.003 IPRD + #.095 xHO2 + #.163 xRCO3 + #.163 xCH2O + #.095 xMGLY + #.264 yR6OOH + #-.575 XC	1.43e-17			
BP55 (New)	RAOOH + OH = #.139 OH + #.148 HO2 + #.589 RO2C + #.124 RO2XC + #.124 zRNO3 + #.074 PROD2 + #.147 MGLY + #.139 IPRD + #.565 xHO2 + #.024 xOH + #.448 xRCHO + #.026 xGLYX + #.030 xMEK + #.252 xMGLY + #.073 xAFG1 + #.073 xAFG2 + #.713 yR6OOH + #1.674 XC	1.41e-10			
BP56 (New)	RAOOH + HV = OH + HO2 + #.5 {GLYX + MGLY + AFG1 + AFG2} + #-.5 XC	Phot Set= MP			
Aromatic VOCs represented in chamber simulations					
BE01 (Updated)	BENZ + OH = #.027 RO2XC + #.31 RO2C + #.57 HO2 + #.31 xHO2 + #.027 zRNO3 + #.57 PHEN + #.31 xGLYX + #.183 xAFG1 + #.127 xAFG2 + #.337 yRAOOH + #.093 OH + #.093 AFG3 + #-0.403 XC	1.22e-12	2.33e-12	0.38	
BE02 (Updated)	TOLU + OH = #.074 RO2XC + #.605 RO2C + #.18 HO2 + #.605 xHO2 + #.074 zRNO3 + #.073 yR6OOH + #.065 xBALD + #.18 CRES + #.29 xGLYX + #.25 xMGLY + #.324 xAFG1 + #.216 xAFG2 + #.606 yRAOOH + #.141 OH + #.141 AFG3 + #-0.176 XC	5.58e-12	1.81e-12	-0.67	
BE03 (Updated)	XYLE + OH = #.098 RO2XC + #.6 RO2C + #.11 HO2 + #.6 xHO2 + #.098 zRNO3 + #.046 yR6OOH + #.04 xBALD + #.11 XYNL + #.11 xGLYX + #.45 xMGLY + #.319 xAFG1 + #.241 xAFG2 + #.651 yRAOOH + #.192 OH + #.192 AFG3 + #.538 XC	2.31e-11			
Steady-State Peroxy Radical operators (for formation of inorganic and radical products)					

RO01 (New)	xHO2 = HO2	k is variable parameter: RO2RO[1]
RO02 (New)	xHO2 =	k is variable parameter: RO2XRO[2]
RO03 (New)	xOH = OH	k is variable parameter: RO2RO
RO04 (New)	xOH =	k is variable parameter: RO2XRO
RO05 (New)	xNO2 = NO2	k is variable parameter: RO2RO
RO06 (New)	xNO2 = XN	k is variable parameter: RO2XRO
RO07 (New)	xMO2 = MO2	k is variable parameter: RO2RO
RO08 (New)	xMO2 = XC	k is variable parameter: RO2XRO
RO09 (New)	xMCO3 = MCO3	k is variable parameter: RO2RO
RO10 (New)	xMCO3 = #2 XC	k is variable parameter: RO2XRO
RO11 (New)	xRCO3 = RCO3	k is variable parameter: RO2RO
RO12 (New)	xRCO3 = #3 XC	k is variable parameter: RO2XRO
RO13 (New)	xMACO3 = MACO3	k is variable parameter: RO2RO
RO14 (New)	xMACO3 = #4 XC	k is variable parameter: RO2XRO
RO15 (New)	xCO = CO	k is variable parameter: RO2RO
RO16 (New)	xCO = XC	k is variable parameter: RO2XRO
Steady-State Peroxy Radical operators (for formation of organic product species)		
PO01 (New)	xCH2O = CH2O	k is variable parameter: RO2RO
PO02 (New)	xCH2O = XC	k is variable parameter: RO2XRO
PO03 (New)	xALD2 = ALD2	k is variable parameter: RO2RO
PO04 (New)	xALD2 = #2 XC	k is variable parameter: RO2XRO
PO05 (New)	xRCHO = RCHO	k is variable parameter: RO2RO
PO06 (New)	xRCHO = #3 XC	k is variable parameter: RO2XRO
PO07 (New)	xACET = ACET	k is variable parameter: RO2RO
PO08 (New)	xACET = #3 XC	k is variable parameter: RO2XRO
PO09 (New)	xMEK = MEK	k is variable parameter: RO2RO
PO10 (New)	xMEK = #4 XC	k is variable parameter: RO2XRO
PO11 (New)	xPROD2 = PROD2	k is variable parameter: RO2RO
PO12 (New)	xPROD2 = #6 XC	k is variable parameter: RO2XRO
PO13 (New)	xMACR = MACR	k is variable parameter: RO2RO
PO14 (New)	xMACR = #4 XC	k is variable parameter: RO2XRO
PO15 (New)	xMVK = MVK	k is variable parameter: RO2RO
PO16 (New)	xMVK = #4 XC	k is variable parameter: RO2XRO
PO17 (New)	xIPRD = IPRD	k is variable parameter: RO2RO
PO18 (New)	xIPRD = #5 XC	k is variable parameter: RO2XRO
PO19 (New)	xRNO3 = RNO3	k is variable parameter: RO2RO
PO20 (New)	xRNO3 = #6 XC + XN	k is variable parameter: RO2XRO
PO21 (New)	xGLYX = GLYX	k is variable parameter: RO2RO
PO22 (New)	xGLYX = #2 XC	k is variable parameter: RO2XRO
PO23 (New)	xMGLY = MGLY	k is variable parameter: RO2RO
PO24 (New)	xMGLY = #3 XC	k is variable parameter: RO2XRO
PO25 (New)	xBACL = BACL	k is variable parameter: RO2RO
PO26 (New)	xBACL = #4 XC	k is variable parameter: RO2XRO
PO27 (New)	xBALD = BALD	k is variable parameter: RO2RO
PO28 (New)	xBALD = #7 XC	k is variable parameter: RO2XRO
PO29 (New)	xAFG1 = AFG1	k is variable parameter: RO2RO
PO30 (New)	xAFG1 = #5 XC	k is variable parameter: RO2XRO
PO31 (New)	xAFG2 = AFG2	k is variable parameter: RO2RO
PO32 (New)	xAFG2 = #5 XC	k is variable parameter: RO2XRO
PO33 (New)	xAFG4 = #6 XC	k is variable parameter: RO2XRO
PO34 (New)	xAFG4 = AFG4	k is variable parameter: RO2RO
PO35 (New)	zRNO3 = RNO3 + #-1 XN	k is variable parameter: RO2NO[3]
PO36 (New)	zRNO3 = PROD2 + HO2	k is variable parameter: RO22NN[4]
PO37 (New)	zRNO3 = #6 XC	k is variable parameter: RO2XRO
PO38 (New)	yROOH = ROOH + #-3 XC	k is variable parameter: RO2HO2[5]
PO39 (New)	yROOH = MEK + #-4 XC	k is variable parameter: RO2RO2M[6]
PO40 (New)	yROOH =	k is variable parameter: RO2RO
PO41 (New)	yR6OOH = R6OOH + #-6 XC	k is variable parameter: RO2HO2

PO42 (New)	yR6OOH = PROD2 + #-6 XC	k is variable parameter: RO2RO2M
PO43 (New)	yR6OOH =	k is variable parameter: RO2RO
PO44 (New)	yRAOOH = RAOOH + #-7 XC	k is variable parameter: RO2HO2
PO45 (New)	yRAOOH = PROD2 + #-6 XC	k is variable parameter: RO2RO2M
PO46 (New)	yRAOOH =	k is variable parameter: RO2RO

1. RO2RO = k(RO2+NO)[NO] + k(RO2+NO3)[NO3] + k(RO2+MCO3){[MCO3]+[RCO3]+[BZCO3]+[MACO3]) + 0.5 k(RO2+MO2)[MO2] + 0.5 k(RO2+RO2){[RO2C]+[RO2XC])
2. RO2XRO = k(RO2+HO2)[HO2] + 0.5 k(RO2+MO2)[MO2] + 0.5 k(RO2+RO2){[RO2C]+[RO2XC])
3. RO2NO = k(RO2+NO)[NO]
4. RO22NN = k(RO2+NO3)[NO3] + k(RO2+MCO3){[MCO3]+[RCO3]+[BZCO3]+[MACO3]) + 0.5 k(RO2+MO2)[MO2] + 0.5 k(RO2+RO2){[RO2C]+[RO2XC])
5. RO2HO2 = k(RO2+HO2)[HO2]
6. RO2RO2M = 0.5 k(RO2+RO2){[RO2C]+ [RO2XC])

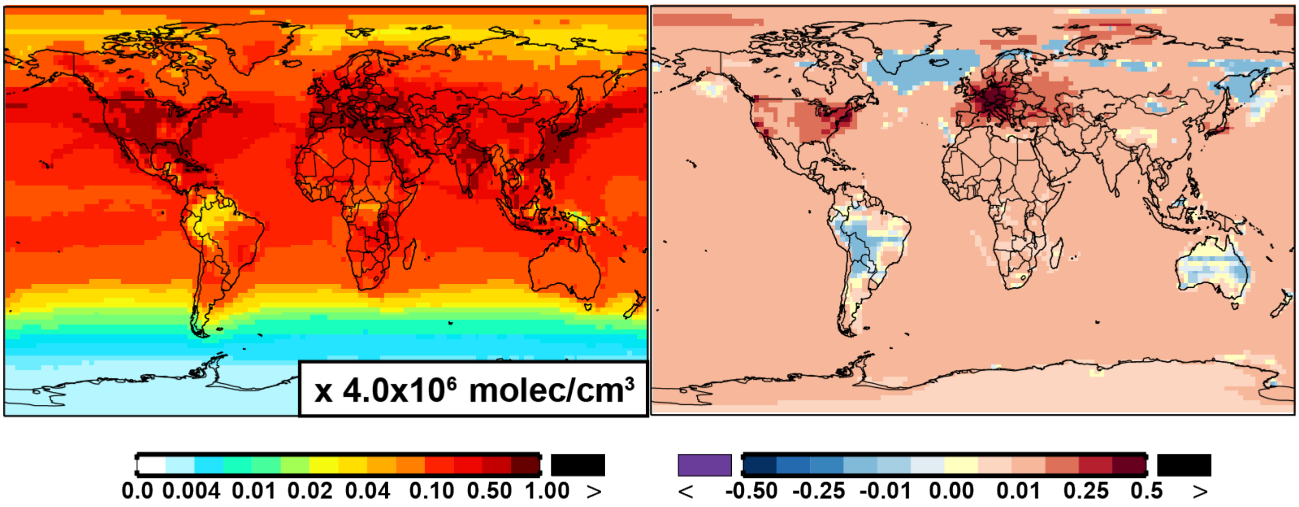

Figure S1. Modeled spatial distributions of surface OH during April−August simulated in the Base case for the year 2005. (Right column) The respective relative changes (%) from Base to SAPRC.
